# Advancements in ecological niche models for forest adaptation to climate change: a comprehensive review

**DOI:** 10.1111/brv.70023

**Published:** 2025-04-03

**Authors:** Wenhuan Xu, Dawei Luo, Kate Peterson, Yueru Zhao, Yue Yu, Zhengyang Ye, Jiejie Sun, Ke Yan, Tongli Wang

**Affiliations:** ^1^ Department of Forest and Conservation Sciences, Faculty of Forestry University of British Columbia, Forest Sciences Centre 2424 Main Mall Vancouver BC V6T 1Z4 Canada

**Keywords:** species distribution models (SDMs), landscape genomics, genecology, forest adaptation, climate niche shift, assisted migration

## Abstract

Climate change poses significant challenges to the health and functions of forest ecosystems. Ecological niche models have emerged as crucial tools for understanding the impact of climate change on forests at the population, species, and ecosystem levels. These models also play a pivotal role in developing adaptive forest conservation and management strategies. Recent advancements in niche model development have led to enhanced prediction accuracy and broadened applications of niche models, driven using high‐quality climate data, improved model algorithms, and the application of landscape genomic information. In this review, we start by elucidating the concept and rationale behind niche models in the context of forestry adaptation to climate change. We then provide an overview of the advancements in occurrence‐based, trait‐based, and genomics‐based models, contributing to a more comprehensive understanding of species responses to climate change. In addition, we summarize findings from 338 studies to highlight the progress made in niche models for forest tree species, including data sources, model algorithms, future climate scenarios used and diverse applications. To assist researchers and practitioners, we provide an exemplar data set and accompanying source code as a tutorial, demonstrating the integration of population genetics into niche models. This paper aims to provide a concise yet comprehensive overview of the continuous advancements and refinements of niche models, serving as a valuable resource for effectively addressing the challenges posed by a changing climate.

## I. INTRODUCTION

Natural selection and local adaptation have historically shaped specific ecological niches for most tree species, allowing them to grow and reproduce within particular habitats across varied geographic landscapes (Vandermeer, 1972; Khatibi & Sheikholeslami, 2016). However, current rapid and unprecedented climate change is disrupting these established distribution patterns, posing critical threats to biodiversity, forest health, productivity, and the provision of ecosystem services (Román‐Palacios & Wiens, 2020; DeMarche, Doak & Morris, 2019). To address these challenges, ecological niche models (ENMs) or niche models, also referred to as species distribution models (SDMs), have emerged as indispensable tools for unravelling the intricate dynamics of forest adaptation to climate change (Barve *et al*., 2011; Schwartz, 2012; Friend *et al*., 1997). With increasing demand and rapid advancements in ENMs (or SDMs), different forms of ecological models have been derived for various data sources, modelling approaches, and model applications. A comprehensive review of the advancements of various ENMs or SDMs could help researchers effectively choose the most appropriate model for their specific objectives.

ENMs have been used to predict suitable habitats at the population, species, and ecosystem levels (Smith *et al*., 2019). Typically, most studies focus on the species level, which may overlook interactions at the ecological community level (Seastedt & Oldfather, 2021) and genetic variation in adaptation to climate change at the population level. In recent years, applications of ecological models at the ecotype level have been used to address the impact of climate change on ecological communities (Hamann & Wang, 2006; Wang *et al*., 2012*b*; Zhang *et al*., 2022). Among‐population variation associated with climate change has been investigated by the application of genecology functions (Hamann & Wang, 2006; Wang *et al*., 2012*b*; Zhang *et al*., 2022) and the integration of landscape genomics, DNA sequencing and genetic markers into niche models (Benito Garzón, Robson & Hampe, 2019; Fitzpatrick & Keller, 2015).

In this context, ENMs can generally be categorized into three main types based on the data that they utilize: occurrence‐based models, trait‐based models, and genomic‐based models. Occurrence‐based models rely on records of species occurrences and typically operate at the species level (Pearson & Dawson, 2003; Araújo & Peterson, 2012). On the other hand, trait‐based and genomic‐based models are often applied at the population level, capturing plasticity and variation within a single species. Trait‐based SDMs (Benito Garzón *et al*., 2019) and genecology models primarily establish relationships between functional traits, such as height, diameter, survival, and environmental conditions at both the planting and seed‐source locations. Genomic‐based models leverage landscape genomic information, like single nucleotide polymorphisms (SNPs), to correlate allele frequencies with environmental conditions (Capblancq *et al*., 2020). DNA sequencing and genetic markers have also been used to improve SDMs at the species level (Ren *et al*., 2018, 2020) and the population level (Bruno Agudo *et al*., 2023; Martínez‐Minaya *et al*., 2019). There is a noted gap in the literature for comprehensive reviews that systematically explore the rationale, advancements, and applications of these three types of niche models, particularly in the context of addressing climate change impacts.

In recent years, the accessibility of extensive species occurrence data and climate data, coupled with advancements in machine learning and geographic information system techniques, has led to a significant increase in studies using niche models to assess the impacts of climate change on forest tree species (Carrillo‐García *et al*., 2023; Gilani *et al*., 2020). However, there is currently a gap in the literature when it comes to summarizing these studies according to the main algorithms they employed, the climate scenarios used, and the versatility of niche models. A comprehensive compilation of these studies is crucial for the advancement of niche model application in forestry practice (Ikeda *et al*., 2017; Zhang *et al*., 2015). Therefore, we searched the literature using the key words: [niche model OR distribution model] AND [tree species] AND [Climate change] in the *Web of Science* and *MEDLINE* (PubMed search engine) databases. We initially collected 3,277 papers, which we screened on October 19th, 2022 by searching for the terms [scenario] OR [projected] OR [prediction] OR [predicted], which yielded a total of 229 studies. From these, we identified 153 papers meeting the following criteria: the paper (*i*) focused on at least one tree species; (*ii*) used distribution models or functions at the species level; (*iii*) provided a climatic range for the targeted species; and (*iv*) predicted future distribution for at least one climate change scenario in at least one future period. As the review process was long, we performed a second search for articles published from October 20th, 2022 to August 1st, 2024, to expand our literature, which added 185 papers to give a total of 338 studies. We recorded the following information from these 338 studies: author, title, published year, abstract, tree species names, model algorithms, future climate data sources, number of climate variables, main content, etc. (see online Supporting Information, Data S1).

This review aims to provide a comprehensive overview of advancements in ENMs and their contribution to understanding forestry adaptations to climate change. We start by discussing the fundamental concepts and rationale behind niche models, highlighting their significance in understanding and mitigating the impacts of climate change on forest populations, species, and ecosystems. We then explore the advancements made in occurrence‐based, trait‐based, and genomics‐based models, shedding light on their potential for advancing niche models. Finally, we investigate the existing literature and analyse the types of models used, study areas, tree species involved, and future climate scenarios employed in niche forest management. Through an in‐depth analysis of the literature, this study contributes to the existing knowledge base and encourages further exploration and refinement of niche models for informed decision‐making in forestry management and conservation practices. The review also includes an exemplar data set with R code for building genomics‐based models, which provides a reference for researchers seeking to understand the influence of climate change on species distribution on population levels and to develop effective forestry strategies.

## II. CONCEPT AND RATIONALE OF ECOLOGICAL NICHE MODELS

### (1) Fundamental niche and realized niche

**Fig. 1:**
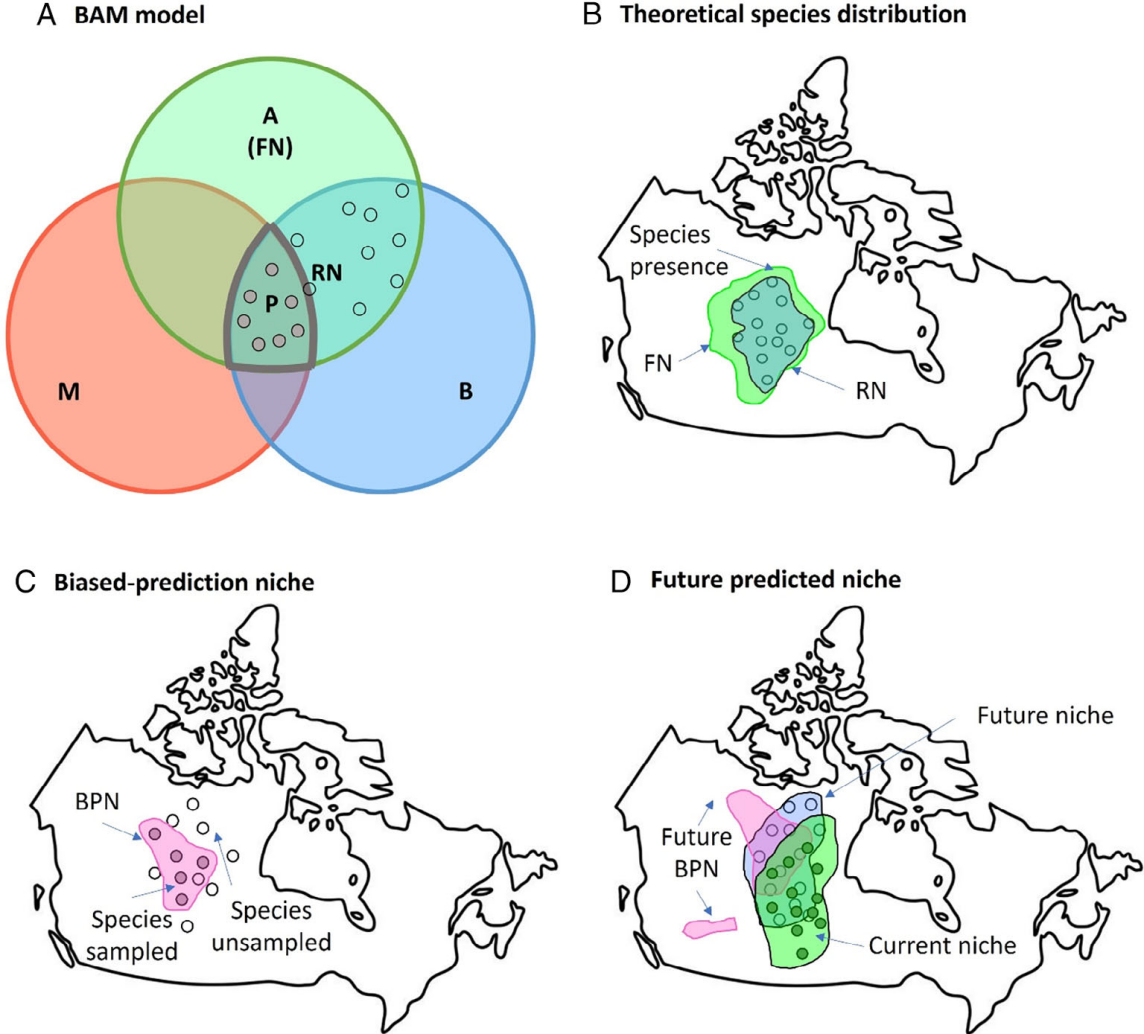
Conceptual graph depicting the concept and rationale of niche models. (A) Biotic–Abiotic–Mobility (BAM) model and concept of fundamental niche (FN) and realized niche (RN) (adapted from Soberon & Peterson, 2005). Green area A represents abiotic factors and can be regarded as the fundamental niche of a species, blue area B represents biotic factors and red area M represents migration capacity of the species. The realized niche can be represented by RN = A∩B. The area P = A∩B∩M represents areas with observed species presence. Dots in the RN area represent species presence points, the grey‐filled dots represent the observed presence points while the empty dots represent the potential species occurrence that has not been observed. (B) The relationship between fundamental niche, realized niche and species occurrence points. (C) The biased prediction niche (BPN, pink region) due to partial occupancy of occurrence points. Filled dots represent species occurrence points that were documented or recorded, while empty dots represent potential occurrence points that have not been observed or sampled. (D) The green area represents the current climate niche for a species; the blue area represents its predicted future niche. The pink area represents the future biased‐prediction niche; as some niche models relate species presence to environmental factors, the future predictions can be outside of the current range.

The fundamental niche (FN) refers to the entire range of conditions in which a species can survive and thrive, independent of biotic interactions and dispersal limitations (Hutchinson, 1957; Holt & Gaines, 1992). By contrast, the realized niche (RN) represents a subset of the fundamental niche where the species actually exists, taking into account historical and current biotic interactions, dispersal limitations, landscape fragmentation, and evolutionary processes (Soberón & Nakamura, 2009). These concepts are foundational to ecological niche theory and can be visualized through a Biotic–Abiotic–Mobility (BAM) diagram (Fig. 1A). The BAM diagram highlights that species distribution is influenced by a combination of abiotic factors (such as climate and soil conditions), biotic interactions (such as competition and predation), and the species' mobility (Soberon & Peterson, 2005).

In the BAM model, region A represents the suitable abiotic conditions for the species, delineating the theoretical boundaries of its fundamental niche, while the realized niche is depicted by the intersection of regions A∩B, representing the area where both abiotic and biotic conditions are favourable for the species' persistence (Fig. 1A). Therefore, the realized niche is a nested subset of the fundamental niche (Fig. 1B), constrained by real‐world ecological factors. For example, in the case of the American chestnut (*Castanea dentata*), its fundamental niche includes a wide range of temperate climates across North America. However, its realized niche has shrunk significantly due to the introduction of the chestnut blight fungus (*Cryphonectria parasitica*), an invasive species that has decimated its populations (Burke, 2012). This illustrates how biotic interactions can dramatically constrain the realized niche relative to the fundamental niche.

ENMs typically predict a species' realized niche using occurrence data from its native range. These models may extend predictions to areas outside the species' current range but within its fundamental niche, revealing potentially suitable habitats that are currently unoccupied due to dispersal barriers or other limiting factors (Pearson & Dawson, 2003). The realized niche is crucial for understanding the current distribution of species, but it might not fully capture the species' potential to adapt to changing conditions. This limitation is particularly relevant in the context of climate change, where predicting future distributions based solely on the realized niche might underestimate a species' capacity for range shifts and overestimate risks in certain areas (Soberón & Nakamura, 2009; Zhao, O'Neill & Wang, 2023). This has been demonstrated for lodgepole pine (*Pinus contorta*) and Douglas fir (*Pseudotsuga menziesii*) in a recent study (Zhao *et al*., 2023). Another factor that should be considered in the context of the BAM framework is human activities, which can alter the mobility (M) of species or lead to new configurations of biotic (B) and abiotic (A) factors (Feng *et al*., 2024).

Estimating the fundamental niche remains a challenge as it requires validation from occurrence and growth data beyond the species' native range. Recent advancements include using a universal response function based on growth traits observed in comprehensive provenance trials to predict the fundamental niche of lodgepole pine (Zhao & Wang, 2023). In provenance trials, populations are often planted outside the species distribution, and models of growth traits can be extrapolated if they are modelled with bell‐shaped curves. In addition, species such as lodgepole pine have been introduced to many countries globally, which can provide useful material for model validation. However, comprehensive provenance trials are scarce which limits the broad application of this approach to other species. Through optimizing modelling algorithms and adjusting cut‐off thresholds, Zhao *et al*. (2023) extended ENMs to predict fundamental niches based on species occurrence data for lodgepole pine and Douglas fir, providing valuable information for developing forestry adaptation and conservation strategies.

### (2) Related terminology

Various terminologies have been used for models developed to study species–environment relationships and predict the realized niche under historical and future climates, including ENMs, SDMs, Bioclimate Envelope Models (BEMs), and Habitat Suitability Models (HSMs). These terminologies are often used interchangeably, although there are subtle differences in their focus and application (Table 1) (Case & Lawler, 2017; Franklin, 2013; Gupta & Sharma, 2019). ENMs typically rely on the relationship between species and their environmental factors, including both biotic and abiotic factors, with a particular emphasis on understanding how these interactions define the niche of a species (Peterson & Soberón, 2012; Wang *et al*., 2016*b*). Climate Niche Models (CNMs) are a subset of ENMs that specifically focus on the impact of climatic variables on species distribution, making them quite similar to BEMs, which also primarily concentrate on the influence of bioclimatic variables (Roberts & Hamann, 2011). SDMs, on the other hand, offer a more specific perspective by incorporating additional factors, including topography, human influences, and dispersal limits to predict species distribution across geographical ranges. HSMs closely align with ENMs in terms of purpose and approach but are more explicitly focused on current habitat conditions rather than the broader ecological niche (Da Re *et al*., 2023). Each of these models, while having overlapping methodologies and goals, is uniquely suited for specific ecological and forestry applications, making them valuable tools for understanding and predicting the impacts of environmental changes on species distributions.

**Table 1:** Comparison of common niche models regarding purpose, approach, application and data input.

Models	Ecological niche models (ENMs), including habitat suitability models (HSMs)	Climate niche models (CNMs), including bioclimate envelope models (BEMs)	Species distribution models (SDMs)
**Purpose**	ENMs predict the potential distribution of species based on their ecological niches, which are the environmental conditions in which a species can maintain a viable population.	CNMs are a subtype of ENMs that specifically focus on climate and aim to predict the species' potential distribution or suitable habitat based on its adaptation to climate variables.	SDMs aim to predict the geographic distribution of a species based on its occurrence and environmental data
**Approach**	ENMs use species occurrence data and environmental variables to model the relationship between species and environmental conditions.	CNMs use climate data as the primary environmental variable to model species distribution.	SDMs can use various types of variables, including climate, soil, and topographic data, and dispersal ability to model species distribution.
**Application**	ENMs are commonly used in biodiversity and conservation studies to assess the impacts of climate change on species distribution, identify suitable habitats for species, and guide conservation planning.	CNMs are widely used in climate change research to estimate the potential range shifts of species under different climate scenarios and to identify areas where species are likely to face suitable or unsuitable climatic conditions in the future.	SDMs are used in a wide range of ecological and conservation applications, including understanding species–environment relationships, predicting potential invasive species' spread, and identifying habitats for conservation.
**Data input**	Species occurrence or presence–absence records and environmental variables (such as climate, soil, topography, etc.).	Climate variables and species presence data.	Species presence data and multiple environmental variables, including abiotic and biotic factors.

For the development and advancement of niche models in forestry applications, it is important to use the correct concepts and terminology (Wang *et al*., 2016*b*; Kearney, 2006). Many papers have used these terms inappropriately, which causes misinterpretation and hinders the full potential of niche models (Peterson & Soberón, 2012). Many studies that use SDMs are actually referring to species niche models (Peterson & Soberón, 2012). In fact, the term SDM strictly refers to models that predict region P in Fig. 1A for the species, i.e. the intersection of regions A∩B∩M. In strict terms, accurately delineating the natural distribution of a tree species is challenging unless all presence points of the species are recorded and collected. When predicting species distribution, many forestry studies are actually predicting potential distributions or suitable habitats based on the accessible presence data of the species (Wang *et al*., 2016*b*), which is the subset of the intersection of regions A∩B, i.e. the realized niche (Fig. 1A). Dispersal limitations are often not considered in forestry studies (Dyderski *et al*., 2018), in which case ENM would be the correct term to use.

### (3) Rationale for future predictions

One of the fundamental rationales for utilizing niche models to predict future species distributions is the concept of niche conservatism, which suggests that species tend to retain their ecological niche over evolutionary time, even in the face of environmental changes (Holt, 1996; Wiens & Graham, 2005). Empirical evidence supports this concept, as demonstrated by Petitpierre *et al*. (2012), who found changes in climatic niches to be rare among terrestrial plant invaders, indicating that many species retain their ancestral climatic niche even when introduced to new environments. By assuming that species maintain their niches in response to climate change, niche models can project potential shifts in species distributions based on their current climatic niche and future climate conditions (Soberón & Nakamura, 2009; Roberts & Hamann, 2011).

Another rationale for using ENMs to predict future changes lies in their capacity effectively to relate current species distribution data to environmental variables, thus providing a predictive framework for how spatial distributions of species' suitable habitats may shift in response to changing environmental conditions (Journé *et al*., 2020). ENMs are particularly valuable in the context of climate change, as they can integrate temporal climate change projections to forecast potential future habitats for species (Soberón & Nakamura, 2009). This modelling approach helps in understanding the likely impacts of climate changes on the adaptation and performance of individual species and the biodiversity of an ecosystem, guiding conservation efforts, and informing strategies for habitat management and species conservation under future climatic scenarios.

When using ENMs for future predictions, it is possible for regions far outside the current distribution range of a species to be predicted as potential future habitats (Dubos *et al*., 2022). For instance, alien species often find suitable habitats in regions far from their native ranges, demonstrating how niche models can predict potential habitats beyond current distributions (Gong *et al*., 2020). To improve the predictive power of niche models, it will be of great benefit to integrate other future environmental variables, such as typology and edaphic factors, as well as population dynamic information, such as population plasticity, competition, migration patterns, and landscape genomics (Waldvogel *et al*., 2020). Edaphic variables, which are expected to remain relatively unchanged in future, are already incorporated into many studies (Ni & Vellend, 2022; Lafleur *et al*., 2010). However, there is ongoing debate regarding this, as some researchers argue that edaphic conditions could change in response to climate change (Pritchard, 2011; Gray & Bishop, 2019), raising questions about the validity of using static soil layers in predicting future species distribution patterns. Despite these controversies, significant advancements have been made in the application of niche models. However, unlike climate variables, future projection data for these factors are unavailable, posing challenges to their direct integration into niche models although some advances have been made (Wu & Nilsson, 2023; Capblancq *et al*., 2020).

## III. ADVANCEMENTS IN OCCURRENCE‐BASED MODELS

**Fig. 2:**
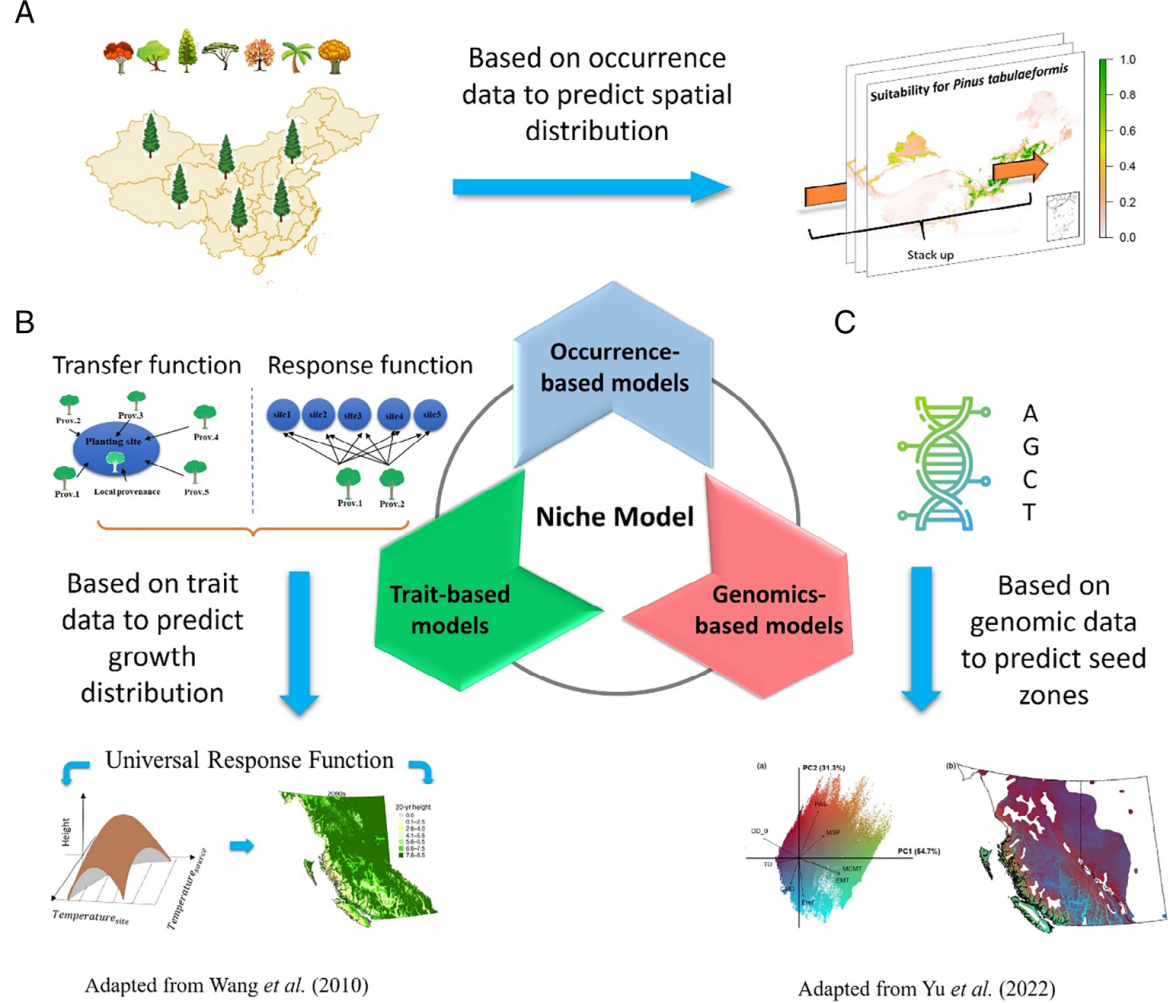
Applications of three types of climate niche models – occurrence‐based, trait‐based and genomic‐based. (A) An occurrence‐based niche model can be used at the community level by stacking the predictive distribution maps for multiple tree species. (B) Example of a trait‐based model where the transfer function and response function are used to predict the universal response function (Wang *et al*., 2010). (C) A genomics‐based model in which information on a single nucleotide polymorphism (SNP) can be applied to delineate seed zones for assisted migration (Yu *et al*., 2022).

ENMs are continuously advancing. The occurrence‐based model is the fundamental approach that utilizes presence–absence data to predict the potential spatial distribution of species (Fig. 2A). This model has a long history and originated from linking the geographic range of species with climate (Flanklin, 1995). Standards for SDMs in biodiversity assessments have been established to emphasize transparency, reproducibility, and rigorous validation to ensure reliable predictions (Araújo *et al*., 2019). Comprehensive guidance on developing HDMs and SDMs using R, from data preparation to model evaluation and application, is provided by Guisan, Thuiller & Zimmermann (2017). The principles and methodologies of predictive habitat distribution models have been extensively reviewed elsewhere, highlighting their application in ecology (Guisan & Zimmermann, 2000). One recent study introduced the ODMAP (Overview, Data, Model, Assessment, and Prediction) protocol, a standardized approach for reporting SDMs (Zurell *et al*., 2020). New approaches have emerged to integrate other biotic and abiotic factors into occurrence‐based niche models, such as incorporating edaphic factors to predict species' future habitats (Sugiyama, 2019; Henneron *et al*., 2020). Besides soil factors, species competition can also affect species niche prediction. One study introduced a novel approach by incorporating niche overlap based on occurrence‐based niche models to integrate interspecific competition (Xu *et al*., 2023). These endeavours highlight the compatibility of the niche model with abiotic (e.g. soil) and biotic (e.g. competition) factors.

Occurrence‐based models can be applied at the community level to predict shifts in ecotype and ecosystems under climate change, providing valuable insight into the dynamics of ecological systems and enabling proactive management and conservation actions to mitigate the impacts of climate change on biodiversity and ecosystem functioning (Jorgenson *et al*., 2015; Ikeda *et al*., 2017). The occurrence‐based model can be applied at the ecosystem classifications (or ecotypes) level, which serves as a framework for forest resources management, by first projecting the potential range shifts of different ecotypes and then associating the ecotypes to individual species (Hamann & Wang, 2006; Wang *et al*., 2012*b*). This information can guide site selection in conservation efforts, allowing assessment of the effectiveness of protected areas (PAs) (Wang *et al*., 2020), and enabling planning of plantation initiatives. One recent study employed this method to investigate the importance of PAs worldwide in providing refuge for over 14,000 species of amphibians and reptiles (Mi *et al*., 2023). Another study effectively forecasted future plant species richness patterns for dryland regions in China (Sun *et al*., 2021*b*).

### (1) Data sources

The quality of occurrence data and climate data has a substantial impact on the accuracy and reliability of ENMs (Dubos *et al*., 2022). Errors in the geographic coordinates of occurrence records can lead to misrepresentation of species distributions and erroneous modelling outcomes. It is therefore crucial to ensure precise and accurate georeferencing of occurrence data. Long‐term data sets spanning multiple years or decades can provide insights into species' responses to environmental changes (Franklin *et al*., 2016; Scherrer *et al*., 2021). Spatial biases, such as uneven sampling efforts across regions or habitats, also can skew species distributions (Fisher‐Phelps *et al*., 2017). Addressing these biases or employing data‐filtering techniques can ensure more representative and unbiased data sets for modelling.

Adequate sample sizes and wide spatial coverage are important for robust modelling. It is essential to represent the full spectrum of environmental conditions encompassed by the species' realized niche, necessitating the collection of as many data points as possible (Sillero *et al*., 2021; Dubos *et al*., 2022). While this is challenging in practice, the rise of global collaboration in biodiversity conservation, technological advancements in digital data management, and the growing appreciation for open‐access scientific data have led to the creation of platforms like the Global Biodiversity Information Facility (GBIF). Established in 2001, GBIF has become the most extensive platform for biodiversity data, offering access to over 1.57 billion specimen occurrences across 53,663 data sets from 1624 data publishers as of July 1, 2020 (Luo *et al*., 2021). GBIF tracks and attributes data usage through DOIs provided to users, while ensuring the reproducibility of research by requiring citations for the use of specific data sets (https://www.gbif.org/citation-guidelines). Furthermore, resources such as the Botanical Information and Ecology Network (BIEN) (https://bien.nceas.ucsb.edu/bien/) significantly enhance the utility of occurrence‐based models by providing extensive data on plant distributions, traits, and taxonomy, which supports detailed analyses and predictions about plant biodiversity under changing climatic conditions. These developments have significantly bolstered the application and effectiveness of niche models.

For climate data, one notable and widely used source is WorldClim, which provides 1‐km resolution global climate data sets (Fick & Hijmans, 2017). Other widely used global data sets such as CHELSA (Karger *et al*., 2017), ERA 5 (Hersbach *et al*., 2020), PRISM (Daly *et al*., 2008), MSWEP (Beck *et al*., 2019), Daymet (Thornton *et al*., 2021) and CliMond (Kriticos *et al*., 2012) also offer high‐resolution climate data that can be essential for accurate climate modelling, particularly in regions where elevation plays a critical role in ecological dynamics. In recent years, to address the need for point‐specific and high‐resolution climate data in mountainous regions, where elevation effects are pronounced, tools such as ClimateNA (Wang *et al*., 2016*a*), ClimateAP (Wang *et al*., 2017) and ClimateEU (Marchi *et al*., 2020) have been developed. These tools offer scale‐free climate data, calibrated with lapse rates to provide tailored climate data for specific sample locations, enhancing the accuracy and applicability of ecological and climatic studies. A recent study suggested that use of ClimateNA is comparable or preferable to employment of onsite micro weather stations (Ye, O'Neill & Wang, 2022). The R package *ClimateNAr* (http://climatena.ca/downloads/ClimateNAr_1.2.0.zip) has been issued recently to enable access to climate data from ClimateNA in R, increasing the effectiveness of incorporating scale‐free climate data in niche models (see Appendix S1 for a step‐by‐step guide and R code). In conclusion, both the quality and resolution of climate data are vital considerations in niche models. Table 2 provides a list of reliable species occurrence and climate data sources used in the studies included in Data S1.

**Table 2:** Sources of species occurrence data and climate data used in the studies reviewed herein.

Type	Data source	Website
Species occurrence data source	Global Biodiversity Information Facility (GBIF)	www.gbif.org
	Integrated Digitized Biocollections (iDigBio)	www.idigbio.org
	National Ecological Observatory Network (NEON)	www.neonscience.org
	National Center for Biotechnology Information (NCBI)	www.ncbi.nlm.nih.gov
	Biodiversity Heritage Library (BHL)	www.biodiversitylibrary.org
	Natural History Museum Data Portal	data.nhm.ac.uk
	DataONE	www.dataone.org
	Global Plant Initiative (GPI)	www.globalplantinitiative.org
	The Plant List	www.theplantlist.org
	Atlas of Living Australia (ALA)	www.ala.org.au
	National Biodiversity Data Centre (NBDC)	www.biodiversityireland.ie
	Consortium of California Herbaria (CCH)	www.cch2.org
	New York Botanical Garden (NYBG) Virtual Herbarium	www.nybg.org/virtual‐herbarium
	The Missouri Botanical Garden (MBG) Tropicos database	tropicos.org
	Royal Botanic Gardens, Kew	www.kew.org/science/data‐and‐resources
	World Flora Online	www.worldfloraonline.org
	Flora of North America	www.efloras.org
	Flora of China	www.efloras.org/flora_page.aspx
	Flora of Australia	www.environment.gov.au/biodiversity/abrs/online‐resources/flora/main
Climate data source	WorldClim	www.worldclim.org
	Climate Data Guide	https://climatedataguide.ucar.edu
	National Centers for Environmental Information (NCEI)	www.ncei.noaa.gov
	NASA Earth Observing System Data and Information System (EOSDIS)	https://earthdata.nasa.gov
	European Centre for Medium‐Range Weather Forecasts (ECMWF)	www.ecmwf.int
	Copernicus Climate Change Service (C3S)	climate.copernicus.eu
	ClimateNA/AP/BC/WNA	www.climatena.ca
	Climate Data Online (CDO)	www.ncdc.noaa.gov/cdo‐web
	Climatic Research Unit (CRU) at the University of East Anglia	www.cru.uea.ac.uk
	Climatologies at High resolution for the Earth's Land Surface Areas (CHELSA)	https://chelsa‐climate.org/
	ECMWF Reanalysis v5 (ERA5)	https://www.ecmwf.int/en/forecasts/dataset/ecmwf‐reanalysis‐v5
	Parameter‐elevation Regressions on Independent Slopes Model (PRISM)	https://prism.oregonstate.edu/
	Multi‐Source Weighted‐Ensemble Precipitation (MSWEP)	https://www.gloh2o.org/mswep/
	Daily Surface Weather Data on a 1 km Grid for North America (Daymet)	https://daac.ornl.gov/DAYMET/guides/Daymet_Daily_V4.html
	Climate Data for Bioclimatic Modelling (CliMond)	https://www.climond.org/
	National Aeronautics and Space Administration (NASA) Earth Observing System Data Gateway	https://earthdata.nasa.gov/eosdis/daacs

### (2) Model algorithms

**Fig. 3:**
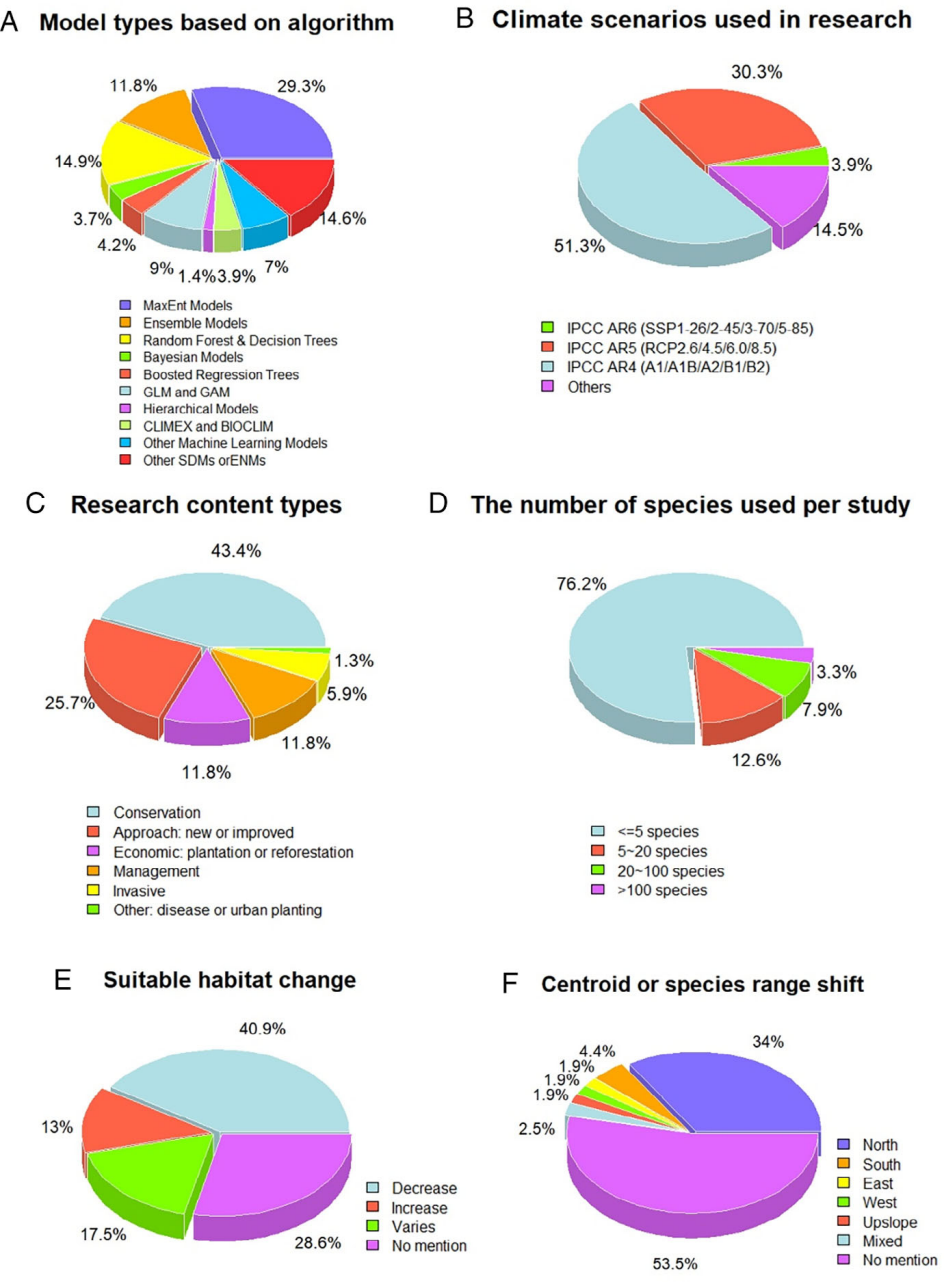
Algorithm, climate scenario, research content, and the number of tree species used in each study for the 338 studies included in the literature review. (A) The model types used, divided into ten categories based on the chosen algorithm. GAM, Generalized Additive Model; GLM, Generalized Linear Model; CLIMEX, mechanistic model; BIOCLIM, correlative model based on bioclimatic envelopes; SDM, Species Distribution Model; ENM, Ecological Niche Model. (B) Climate scenarios used in research. (C) Research content categorized into six types. (D) The number of tree species used in each study. (E) Suitable habitat change in future; ‘decrease’ and ‘increase’ represent predicted future suitable habitat loss and expansion respectively, ‘varies’ indicates where the predicted trend includes both increase or decrease among different species or different periods. (F) Direction of centroid shift or species range shift in future; ‘upslope’ represents higher elevation, ‘mixed’ indicates that different directions were predicted across different species or different periods.

The advancement in machine‐learning algorithms has significantly enhanced niche model applications by enabling more sophisticated modelling approaches and improving prediction accuracy (Phang *et al*., 2023; Wiley *et al*., 2003). Machine‐learning techniques, such as Random Forests (RF), Maxent, and Neural Networks (NN), can handle complex relationships between species distributions and environmental variables, leading to improved modelling performance and a better understanding of species' responses to changing climates (Young, Carter & Evangelista, 2011; Warren & Seifert, 2011; Breiman, 2001). These algorithms have created new opportunities for integrating extensive data sets and diverse environmental variables, resulting in more comprehensive and adaptable niche models (Elith *et al*., 2006). Based on the 338 studies in our literature review, Maxent was the most frequently utilized algorithm, with nearly 30% of the studies employing this method (Fig. 3A). Maxent is known to be capable of handling presence‐only data and can capture complex relationships between species occurrences and environmental variables (Booth *et al*., 2014*b*). However, it is important to note that Maxent can be difficult to parameterize and tends to overpredict, which could affect the model's accuracy in certain applications, such as in studies assessing model performance across different temporal contexts using palaeobotanical data (Moreno‐Amat *et al*., 2015). RF or decision tree‐based models were also commonly utilized, with nearly 15% of studies employing these algorithms (Fig. 3A). RF models are popular due to their robustness, ability to handle interactions among variables and advantages for overcoming collinearity and overfitting (Breiman, 2001; Cutler *et al*., 2007). Ensemble models, which combine multiple modelling techniques, have increased in popularity. These models enhance the robustness of predictions by integrating diverse perspectives, thereby mitigating to some extent the uncertainties associated with individual models. The advantages of such integrative approaches are further highlighted by the use of ensemble forecasting, which exemplifies how combining different models can lead to more reliable predictions (Araújo & New, 2007). Supporting this approach, Marmion *et al*. (2009) provide a critical evaluation of consensus methods, underscoring the value of ensemble approaches in SDMs.

Techniques such as hierarchical modelling and Bayesian statistics have emerged as powerful tools for dissecting complex ecological data (Sun *et al*., 2021*b*). Hierarchical models allow for the integration of data across different scales, thus accommodating variability from local to regional levels. This approach was demonstrated in studies by Mateo *et al*. (2019), which explored optimal hierarchical methods for meaningful niche modelling and emphasized their importance in vegetation conservation at the landscape scale. Bayesian methods, on the other hand, offer robust statistical frameworks for incorporating uncertainty and prior knowledge into SDMs, allowing for more detailed and informed predictions. These methods have been pivotal in refining predictions of species vulnerability to climate change (Scherrer *et al*., 2021), and in adjusting models to account for niche truncation, which can significantly improve spatial and temporal predictions of species distributions (Chevalier *et al*., 2022).

The advancement of algorithms has been greatly supported by software packages designed to handle complex ecological data. One such package is hSDM, an R package that utilizes hierarchical Bayesian models to estimate parameters from species occurrence and abundance data (Vieilledent *et al*., 2023). This package enables the analysis of multiple hierarchical processes, such as habitat suitability and spatial dependence, enhancing our understanding of ecological patterns (Royle & Dorazio, 2008). Another robust tool, BIOMOD2, also developed for R, excels in ensemble forecasting of species distributions (Thuiller *et al*., 2016). It supports a variety of modelling techniques including generalized linear models, generalized additive models, and machine learning methods like RF and Maxent (Thuiller *et al*., 2009). Additionally, IBIS.iSDM represents a newer development in this field, offering an integrated framework that combines various data sources for SDMs (Jung, 2023). It accommodates different types of data input, from presence‐only data to community surveys and expert evaluations of species ranges. The prevalence of these different modelling algorithms and software showcases the versatility and flexibility of niche model approaches, allowing researchers to select the most suitable method based on their specific research objectives (Ferrarini *et al*., 2019).

### (3) Advances in future climate scenarios used

IPCC Assessment Reports (ARs) are prominent sources of climate change scenarios used in niche model studies (Fig. 3B) (IPCC, 2018; Cramer *et al*., 2001). The latest report, IPCC AR6, which introduced the Shared Socioeconomic Pathways (SSP1‐26, SSP2‐45, SSP3‐70, SSP5‐85), differs from previous ARs by providing more up‐to‐date and comprehensive assessments of climate science, social‐economic impacts, and mitigation strategies (Ming *et al*., 2021). It incorporates the latest scientific findings and advances in climate modelling and improves the accuracy of climate projections. The use of IPCC AR6 data in niche model applications allows for more accurate predictions of species distributions under future climate scenarios, enabling better‐informed conservation and management decisions (Nicholls *et al*., 2022). AR6 and IPCC AR5 (RCP2.6, RCP4.5, RCP6.0, RCP8.5), were utilized in around 34% of the studies included in our literature review. The earlier IPCC AR4, including scenarios A1(A1B), A2, B1, and B2, was employed in over half of the studies. These findings highlight the reliance on IPCC reports for future climate projections in SDMs and niche model studies (Meinshausen *et al*., 2011; Goberville *et al*., 2015).

The uncertainty of future climates poses challenges in predicting the ecological impacts of climate change and managing natural resources sustainably (Pecchi *et al*., 2019; Gregor *et al*., 2022). Current projections often rely on a single mid‐range climate change scenario or a limited number of combinations of global climate models and greenhouse gas emissions scenarios. While these approaches simplify interpretation, they can lead to biased projections and fail to consider the full range of plausible future climates. Some researchers have proposed using the average of future climates (Jackson, Meister & Prudhomme, 2011) or ensembles (Mahony *et al*., 2022), or a ‘consensus’ approach, which takes consensus model predictions using different climate scenarios, to avoid the loss of spatial and temporal variation inherent in each climate scenario (Wang *et al*., 2012*b*). This method provides a more reliable representation of the most frequently projected ecosystem climates across all scenarios, offering a more informed response to future climate uncertainty.

## IV. ADVANCEMENTS IN TRAIT‐BASED MODELS

**Fig. 4:**
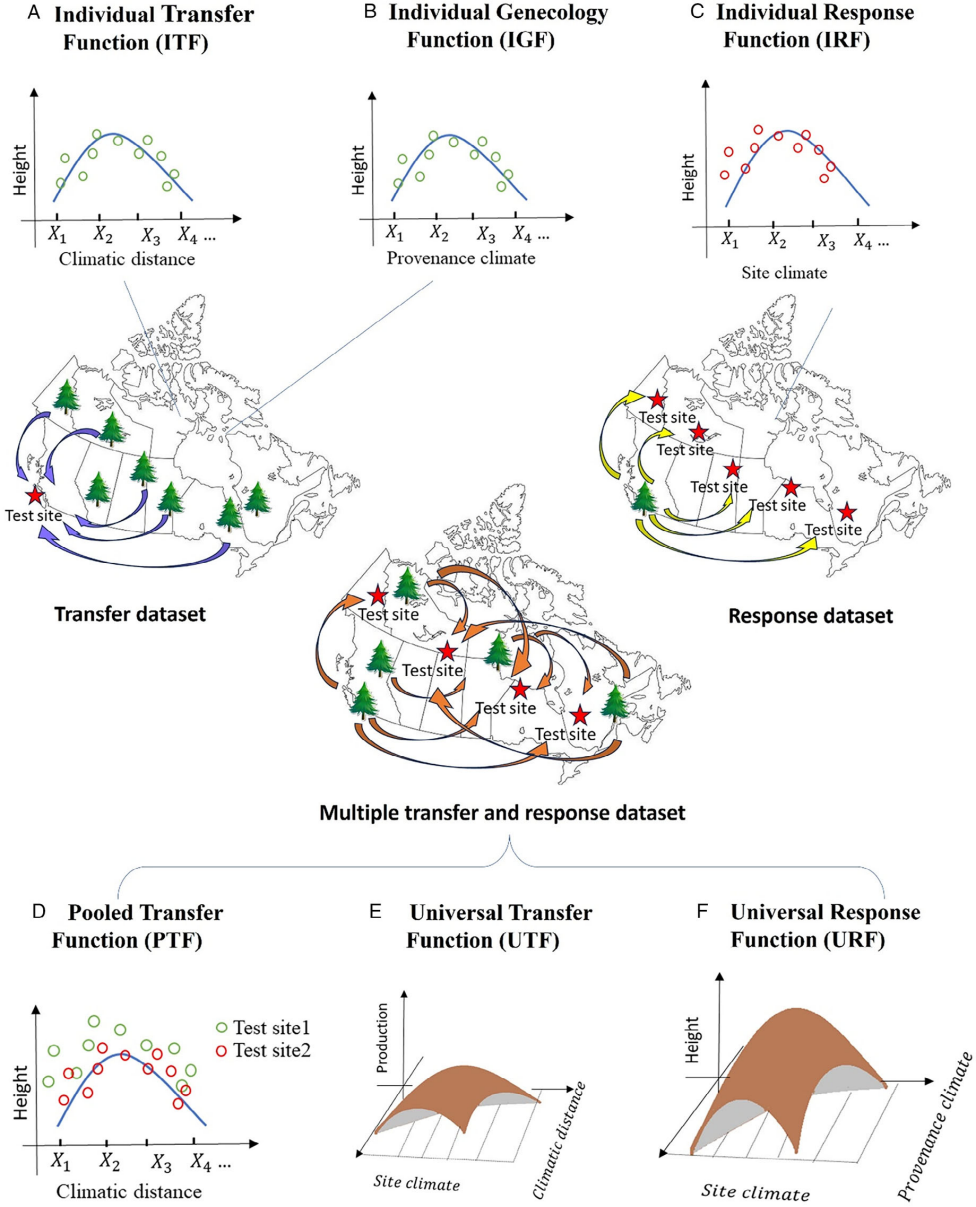
The development of trait‐based models and the key functions. All the functions are based on transfer or response data sets. The transfer data set involves diverse populations transferred to a single test site, while the response data set comprises a single population tested across multiple sites. (A) Individual Transfer Function (ITF) relates populations' fitness (e.g. height) to the climatic distance between provenance climates and a test site; (B) Individual Genecology Function (IGF) relates populations' fitness to provenance climates within one test site; (C) Individual Response Function (IRF) relates the fitness of one population to climates in multiple test sites; (D) Pooled Transfer Function (PTF) is an ensemble of ITFs across two or more test sites; (E) Universal Transfer Function (UTF) relates populations' fitness to both climate distance and test site climates; (F) Universal Response Function (URF) links populations' fitness to climates in both test sites and provenance locations. Each tree in the map symbolizes a population or provenance, while differently coloured circles in the plots depict distinct test sites.

The second type of model is the trait‐based model. These models are constructed based on the fitness traits of populations, such as height or survival, measured in provenance tests or common‐garden experiments, and the climate gradients associated with the population sources (Benito Garzón *et al*., 2019). Initially, rather than predicting species distribution or geographic range, these models were intended for finding optimal populations for planting or safe‐seed transfer zones and seed zone delineation through the relationship between phenotypic traits of populations and climate conditions (Raymond & Lindgren, 1990; Mátyás, 1994; Parker & Niejenhuis, 1996). Individual Transfer Function (ITF) was proposed to understand population variation by linking variation in fitness traits to transfer distances in a specific site (Fig. 4A) (Mátyás, 1994). In comparison, Individual Response Function (IRF) focuses on the effects of environmental gradient on fitness traits for a specific population (Fig. 4C) (Rehfeldt *et al*., 1999). The use of IRFs has been improved by a multivariate approach incorporating climate variables into a single model (Wang *et al*., 2006). In addition, using climates of their source locations, the Individual Genecology Function (IGF) was developed for use with the same data set to explore the different performance of populations at a given test site (Fig. 4B) (Wang *et al*., 2006; St. Clair *et al*., 2022). By pooling data from multiple test sites, a Pooled Transfer Function (PTF) or a general transfer function was built (Fig. 4D) (Rehfeldt *et al*., 1999). To improve prediction accuracy, methods for standardizing site effects on productivity were applied to help guide seed transfer practices and to help establish climate‐based seed transfer systems (Schmidtling, 1994; O'Neill *et al*., 2017). The general transfer functions assume identical response curves across populations for each site, which is often unrealistic. To overcome this limitation, the Universal Transfer Function (UTF) was developed, linking population fitness traits to both climate transfer distance and site climate, therefore demonstrating population differences in response to climate change (Fig. 4E) (O'Neill, Hamann & Wang, 2008). Similarly, to enhance understanding of how different populations respond to climate change, the Universal Response Function (URF) was proposed to incorporate environmental effects (plasticity) and genetic effects (local adaptation) into a single model that can predict the growth potential of any population at any planting site (Fig. 4F) (Wang, O'Neill & Aitkin, 2010), which was achieved by including multiple climate variables that describe the population origin and the planting site. URFs have provided successful predictions of future growth potential for species such as *Pseudotsuga menziesii* (Chakraborty *et al*., 2015), *Pinus strobus* (Yang *et al*., 2015), *Platycladus orientalis* (Hu *et al*., 2019), and *Picea abies* (Liepe *et al*., 2022). In particular, a URF was applied to predict the global fundamental niche of *Pinus contorta* (Zhao & Wang, 2023).

These trait‐based models offer valuable insights for developing forestry measures in response to climate change as they integrate the plasticity and adaption of populations into the conventional niche model framework (Benito Garzón *et al*., 2019). This integration allows for a more comprehensive understanding of the adaptive variation of a species and enhances the accuracy of predictions in a changing climate. By incorporating the plasticity and adaptation of populations, trait‐based models provide valuable tools for forestry practitioners to make informed decisions and develop adaptive management strategies in the face of changing climatic conditions (Niklaus *et al*., 2017; Abakumova *et al*., 2016).

## V. ADVANCEMENTS IN GENOMICS‐BASED MODELS

The third type of model is the genomics‐based model, also known as the landscape genomics model. It integrates genetic and genomic information into traditional niche modelling and offers valuable insights for niche predictions (Wu & Nilsson, 2023). Recent studies have shown that integrating genetic markers into SDMs can provide critical insights into plant diversification and species distributions in response to climate change (Gotelli & Stanton‐Geddes, 2015, Bruno Agudo *et al*., 2023). For example, research on primula species in southwest China and the Qinghai‐Tibet Plateau revealed key insights into their demographic history and divergence (Ren *et al*., 2018, 2020). For *Primula tibetica*, this approach demonstrated the genetic consequences of Quaternary climatic oscillations (Ren *et al*., 2017). One widely used approach to construct a genomics‐based model at the population level involves using allele frequencies of SNPs as dependent variables and climate variables of population origins as independent variables, assuming local adaptation of these populations. This type of genomics‐based model has practical applications in the development of seed and breeding zones for species lacking long‐term common garden data, and in the prediction of seed and breeding zone shifts under future climates, providing valuable insights for vulnerability ranking of widespread species in the context of climate change (Yu *et al*., 2022). It can also provide information to calculate genetic offsets, which reflect the genetic distance or divergence (based on allele frequency) between current and future environmental conditions (Rellstab, Dauphin & Exposito‐Alonso, 2021). It provides valuable information on the adaptive potential and vulnerability of tree populations to climate change.

The gradient forest (GF) algorithm has been used frequently to develop landscape genomics models, but was initially developed to explore the complex non‐linear relationships between species abundance and environmental gradients at the community level (Ellis, Smith & Pitcher, 2012). GF algorithms were later adapted for use with SNP data to characterize non‐linear local adaptation patterns and identify important environmental variables that drive local adaptation (Fitzpatrick & Keller, 2015; Capblancq *et al*., 2020). Additionally, GF models provide an opportunity to measure the extent to which the gene–environment relationship is likely to be interrupted (i.e. genetic offset), and to assess the vulnerability of a population to climate change (Fitzpatrick & Keller, 2015; Capblancq *et al*., 2020).

To illustrate the construction of a genomic‐based niche model, we utilized a lodgepole pine sampler data set to build a GF model (Mahony *et al*., 2020). For genomics input data, we used a total of 32,407 SNPs from 281 lodgepole pine populations (Appendix S2), consisting of 1,907 individual lodgepole pine trees across British Columbia and Alberta in western Canada (Appendix S3 and S4, see also Figs S1 and S2 in Appendix S1). Environmental data for 20 climate variables were generated *via* the recently released *ClimateNAr* package (Steps 1–3 and Table S1 in Appendix S1) to construct the final GF model (Steps 4–9 and Figs S3–S5 in Appendix S1).

**Fig. 5:**
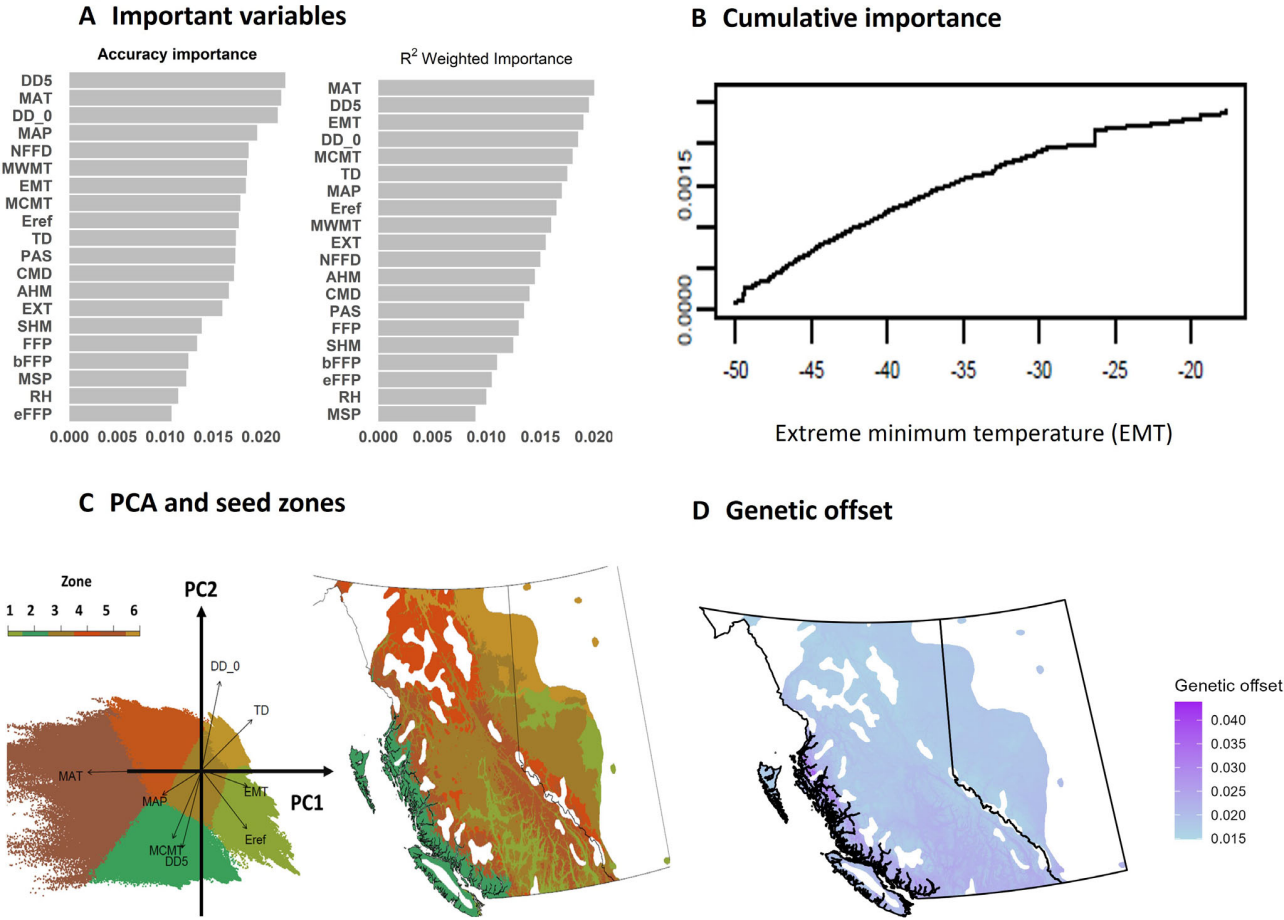
Application of a gradient forest climate niche model to data for lodgepole pine in response to climate change. (A) Ranking of 20 climate variables driving the local adaptation pattern across 281 lodgepole pine populations in British Columbia and Alberta, Canada. See Table S1 for definitions of climate variables. (B) Cumulative importance plot showing the significant allele frequency change for extreme minimum temperature (EMT). (C) Seed zones cluster based on principal component analysis (PCA) results shows the correlation between allele frequency and environmental variables. (D) Genetic offsets of lodgepole pine under the predicted climate RCP8.5 2041–2070.

The trained GF model outputs two key pieces of information: important local adaptation drivers and non‐linear allele frequency changes along these drivers. The predictor importance graph indicates each climate variable's contribution to the species' local adaptation (Fig. 5A) and the cumulative importance plot (Fig. 5B) reveals allele frequency changes across important environmental variables (Fitzpatrick & Keller, 2015; Ellis *et al*., 2012). For example, in our data set, cumulative importance for extreme minimum temperature (EMT) consistently increased, with a notable change occurring at an EMT of −26 °C (Fig. 5B). This suggests significant genotype variations between populations situated below and above this threshold. Using principal component analysis (PCA), we clustered all the locations inside the lodgepole pine distribution range according to the pattern of genomic variation, enabling the delineation of seed zones (Fig. 5C, Figs S6–S10 in Appendix S1).

We conducted a genetic offset analysis using climate data from both the reference period and the 2041–2070 Representative Concentration Pathway (RCP) 8.5 scenario (Fig. 5D, see Section 6 and Figs S11 and S12 in Appendix S1). Our findings indicate that populations in southern and coastal British Columbia may experience higher climatic stress under projected climate change (Aguirre‐Liguori, Ramírez‐Barahona & Gaut, 2021). Overall, the integration of genomics information into niche models represents a promising direction for advancing our understanding of the adaptive capacity of tree populations and their responses to changing environmental conditions (Rellstab *et al*., 2021; Capblancq *et al*., 2020).

## VI. FORESTRY APPLICATIONS

Niche models are applied in forestry in increasingly diverse ways that encompass conservation, management, and economic considerations (Fig. 3C) (Saenz‐Romero *et al*., 2015). Around 43% of papers designed their study for conservation purposes, highlighting the importance of understanding species distributions for conservation planning and decision‐making. Another clear focus was the development of new approaches or methodological improvements to obtain higher predictive power and refinement of modelling techniques (Ferrarini *et al*., 2019). Economic aspects such as plantation or reforestation were also a significant focus, demonstrating the relevance of niche models for informing sustainable land use and economic development (Fig. 3C). Management‐related studies (11.8%) emphasized applications of niche models in guiding effective management strategies. A smaller proportion of studies used niche models to understand and mitigate challenges associated with invasive species, diseases, and effects of urban planting etc (Verhoeven, Glisson & Larkin, 2020).

Most studies in our literature review focused on a relatively small number of tree species, with five or fewer species included in 76% of analyses (Fig. 3D). This suggests a relatively targeted approach, potentially focusing on specific ecological or conservation priorities (Schueler *et al*., 2014). A smaller proportion of studies (12.6%) included a moderate number of species, ranging from five to 20. These observations underscore the importance of conducting more comprehensive assessments that involve multiple tree species, thereby enabling a more intricate exploration of the collective responses of tree species to climate change, encompassing aspects like species richness and composition (Dyderski *et al*., 2018).

Our literature review also revealed a significant trend towards habitat loss, with over 40% of studies predicting a decrease in suitable habitat, indicating that climatic shifts are likely impacting habitats negatively (Fig. 3E). Conversely, only 13% of studies forecast an increase in suitable habitats, representing new opportunities for some species adapting to changing conditions. Furthermore, 17.5% of the studies reported mixed results, illustrating varied ecological responses across different species and time periods. A notable 28.6% of studies did not specify habitat changes, focusing more on model performance comparisons, new methods, or other aspects. Directional shifts in species distribution, where mentioned, revealed a predominant movement northward, observed in 34% of studies, likely as species migrate towards cooler, more temperate climates due to global warming (Fig. 3F). By contrast, reports of shifts to the south, east, west, or upslope were rare (<10%), and a small fraction (2.5%) reported mixed directional shifts. A significant 53.5% of studies did not specify any direction of range shift, potentially highlighting a focus on broader distribution changes or methodological aspects. These insights may be crucial for shaping effective conservation strategies and forestry management, emphasizing the need for adaptive management that considers the predicted northward and upslope shifts (Srivastava, Lafond & Griess, 2019).

**Fig. 6:**
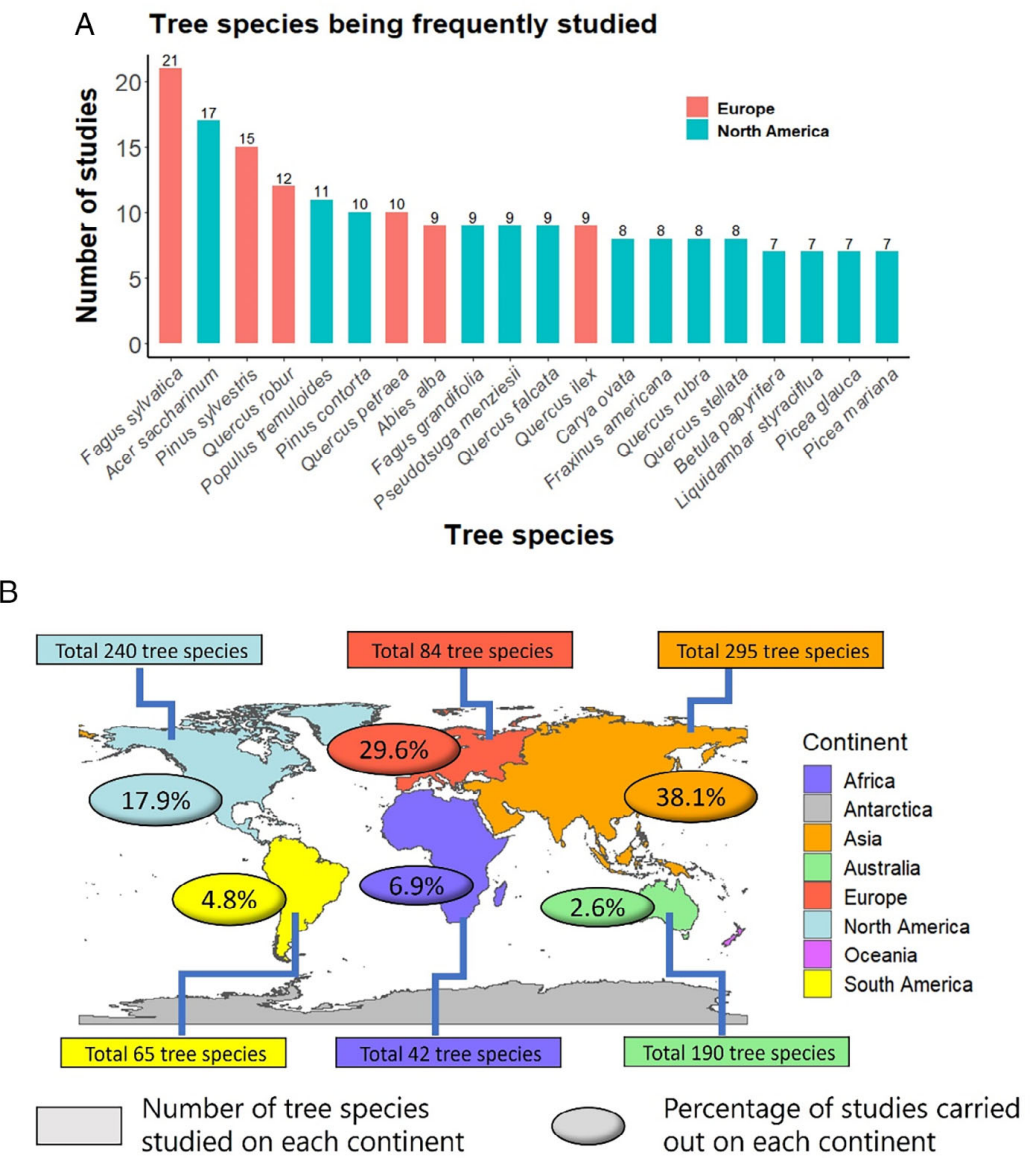
(A) The 20 tree species studied most frequently in our database. (B) The number of tree species studied on each continent and the percentage of studies for each continent (*N* = 338 studies) included in the literature review.

We identified the 20 tree species that have been studied most extensively within the 338 research papers in our database (Fig. 6A). *Fagus sylvatica* and *Acer saccharum* (sugar maple) have received the most attention, with 21 and 17 studies, respectively, indicating their ecological importance and the wide availability of data for these species (Xie, Civco & Silander, 2018). The prominence of these species may result from multiple factors such as ecological significance and economic value, but should not overshadow the importance of understanding and conserving other tree species, especially rare or endangered species, for which data availability may be limited. Further research efforts should aim to include a broader range of tree species to ensure comprehensive insights into the distribution and responses of other tree species to climate change (Guisan *et al*., 2006; Galatowitsch, Frelich & Phillips‐Mao, 2009).

We also mapped the distribution of studies across different continents (Fig. 6B). Asia has the highest proportion of studies, with 38.1% of publications, followed by Europe and North America with 29.6% and 17.9% of studies, respectively. When considering the number of tree species studied within each continent, Asia again stands out with 295 different species of tree investigated in niche model studies, followed by North America (240), Australia and Oceania (190), and then Europe (84). These findings highlight a global distribution of research efforts involving niche models. However, further exploration and research in Africa and South America is needed, as fewer studies on a smaller number of species have been conducted on these continents (Kooperman *et al*., 2018; Fraser *et al*., 2013).

## VII. DISCUSSION AND FUTURE DIRECTIONS

ENMs have emerged as valuable tools in forestry applications to address the challenges posed by climate change (Wang *et al*., 2012*b*; Tiansawat, Elliott & Wangpakapattanawong, 2022). Using the niche model, researchers and forest managers can assess potential impacts of climate change on forests at the scale of population, species, and ecosystems, as well as develop strategies to mitigate these impacts (Xu & Prescott, 2024). The advancement of ENMs has enabled a better understanding of the complex relationships between climate variables and species distributions, allowing for more accurate predictions and informed decision‐making (Ferrarini *et al*., 2019). One key advancement of niche models is the development of sophisticated machine‐learning algorithms, such as Maxent, RF, and hierarchical approaches, which have enabled the use of large data sets and enhanced the predictive power and robustness of niche models. These machine‐learning algorithms have been applied to optimize sampling strategies for plant species distribution at landscape scales (Mateo *et al*., 2018) and to select suitable species for forest restoration (Gastón *et al*., 2014). The significant enrichment of plant occurrence data and improved accessibility and accuracy of climate variables have also contributed to recent advancement in ENMs. Advancements in trait‐based models, particularly provenance‐test‐based transfer and response functions have enabled ENMs to address among‐population variation within a species. Furthermore, the incorporation of genomic data (Yu *et al*., 2022) and other environmental variables, such as soil data, has further improved the accuracy and applicability of niche models in forestry applications. These machine‐learning algorithms have been applied to form adaptive management strategies, including assisted migration and informed reforestation practices, to promote the resilience of tree species in the face of climate change and prioritize conservation efforts (Barragán, Wang & Rhemtulla, 2022; Aitken & Bemmels, 2016). However, it is important to acknowledge the limitations and uncertainties associated with niche models. Data quality, model assumptions, and the availability of long‐term climate projections can influence the accuracy of predictions.

Continued research on modelling techniques and data collection efforts are needed to improve model performance for forestry resource management in changing climates. Future research directions include, but are not limited to, the following aspects.

(1) Future research should explore the projection of environmental variables other than climate, especially those influenced by climate change, like soil properties. Generating future projections for these variables is challenging yet essential for improving the predictive power of species niche models. Innovative approaches to integrating these environmental variables into niche models warrant investigation (Xu *et al*., 2023).
(2) Integrating biotic factors into niche models is crucial. While some areas may be predicted as suitable habitats under future climate scenarios, competition from other species can alter this suitability (Xu *et al*., 2023; Moran & Ormond, 2015). Quantifying competition levels and their impact on species presence probability will be an important focus of future efforts. Additionally, for species reliant on insect or animal movement for regeneration or migration, predicting the effects of climate change on these biotic interactions and integrating them into niche predictions requires further research (HilleRisLambers *et al*., 2013; Van der Putten, Macel & Visser, 2010).
(3) To enhance niche predictions for individual plant species, models should consider ecotype and population‐level adaptations (Smith *et al*., 2019). When assessing a location's suitability for a particular species, we must also consider the surrounding context. For instance, if a site is projected to be suitable for one species but its surroundings are expected to transition into non‐forested ecosystems in the future, those locations should be deemed unsuitable. Additionally, certain tree species exhibit variations in allele frequencies across landscapes. These species may adapt to new climates through adjustments in allele frequencies within populations or through gene flow (Aguirre‐Liguori *et al*., 2021). Quantifying the impacts of these genetic adaptations and estimating the affected habitat areas remain areas of uncertainty that require further investigation.
(4) Many traditional niche models have relied on ArcGIS through the Maxent algorithm, and are often constrained by their ability to incorporate only 19 or fewer climate variables due to computational limitations and system settings. Nevertheless, there exist more than 70 annual, seasonal, or monthly climatic variables, and different species may exhibit sensitivity to distinct variables (Zhao *et al*., 2022; Wang *et al*., 2022). Another computational bottleneck arises when constructing niche models for a large number of species with extensive occurrence data. This constraint has historically led to the prediction of niche shifts for only one or a few species in most studies (Dyderski *et al*., 2018). However, expanding our capabilities to predict the niches of multiple species and integrating them can significantly enhance our understanding of species richness, diversity, and composition, enabling the exploration of more intricate ecological questions (Gilani *et al*., 2020; Sun *et al*., 2021*a*). Hence, it is imperative to invest in research aimed at refining machine‐learning algorithms and streamlining the workflow of niche model development to accommodate a larger number of species, extensive occurrence data, and multiple climate or non‐climatic variables.
(5) While most niche models have traditionally been developed from a species perspective, a critical shift is needed towards applying these models more effectively in forestry practices. Many aspects of forestry, including reforestation, ecological restoration projects, and plantation management, require tools or models that are site‐based (Liepe *et al*., 2016; Harrison *et al*., 2017). For instance, identifying suitable tree species that can thrive in both current and future climates is a pivotal step in reforestation efforts, and this process is greatly facilitated by site‐based models. However, most previous ENMs have predominantly focused on predicting where species will be suitable or vulnerable in the future, leaving a void in the provision of site‐based models that can guide the selection of appropriate tree species for specific planting locations. To bridge this gap between theoretical modelling and on‐the‐ground application, it is imperative to pivot towards the development of niche models that cater to site‐based forestry needs, enhancing their practical utility in various forestry endeavours.

Overall, the advancements in three types of niche models – occurrence‐based, trait‐based, and genomics‐based – have made substantial contributions to our comprehension of climate change impacts on tree species and ecosystems within the realm of forestry applications. These models hold the promise of evolving further, ultimately serving as indispensable decision‐support tools for forest managers and policymakers (Ikeda *et al*., 2017; Gong *et al*., 2020). Their continued development will empower the formulation of proactive and adaptable strategies to counteract the adverse impacts of climate change on forests, thereby fostering their enduring sustainability and vitality (Journé *et al*., 2020; Ikeda *et al*., 2017).

## VIII. CONCLUSIONS

(1) Ecological niche models (ENMs) are powerful tools for understanding and addressing climate change impacts on forests, particularly through advancements in machine learning algorithms, trait‐based models, and genomics techniques.
(2) Significant advancements in ENMs include the development of sophisticated machine‐learning algorithms (e.g. Maxent, RF), trait‐based models incorporating provenance‐test‐based transfer and response functions, and the integration of genomic data and other environmental variables, all of which enhance predictive power and address among‐population variation.
(3) Future research should prioritize the integration of non‐climatic environmental variables, biotic interactions, and population‐level genetic adaptations to enhance the accuracy and applicability of ENMs in forestry.
(4) Expanding ENMs from species‐based predictions to site‐based models is crucial for translating theoretical ENM outputs into practical forestry applications such as reforestation and ecological restoration.

## Supporting information


**Data S1.** Full list of articles identified by our search and information derived from each article.

**Appendix S1.** R tutorial for creating a genomic‐based niche model.

**Appendix S2.** Geographic location information of the 281 lodgepole pine populations.

**Appendix S3.** Shapefile for the range of lodgepole pine in British Columbia and Alberta.

**Appendix S4.** Outline of the political boundary of British Columbia and Alberta.
