# Supplementary material for: Advancements in ecological niche models for forest adaptation to climate change: a comprehensive review
Source: Biol Rev Camb Philos Soc. 2025 Apr 3;100(4):1754–81. doi: 10.1111/brv.70023 (PMC12227795; doi:10.1111/brv.70023)
Supplement: Supplementary file 2 — Appendix S1. R tutorial for creating a genomic‐based niche model. [file BRV-100-1754-s003.docx]

**Appendix S1. R tutorial for genomic based model**

Contents

([1) Species data description 2](#_Toc147221102)

[Provenance trial 2](#_Toc147221103)

[Genomics data 3](#_Toc147221104)

([2) Scale-free climate data generation 5](#_Toc147221105)

[Step 1. ClimateNA application download 5](#_Toc147221106)

[Step 2. ClimateNAr package installation 5](#_Toc147221107)

[Step 3. Climate data generation for 281 locations 5](#_Toc147221108)

([3) Genomic data preparation 7](#_Toc147221109)

[Step 4. Convert the raw SNP data to frequency format 7](#_Toc147221110)

[Step 5. Discard SNPs with minor allele frequency < 0.05 8](#_Toc147221111)

[Step 6. Discard SNPs with > 30% missing data 8](#_Toc147221112)

(4) [Gradient forest model 8](#_Toc147221113)

[Step 7. Merge genomic data and climate data 9](#_Toc147221114)

[Step 8. GF model training 9](#_Toc147221115)

[Step 9. GF model output plot 10](#_Toc147221116)

(5) [Geographic plotting 13](#_Toc147221117)

[Step 10. Map projection 13](#_Toc147221118)

[Step 11. Stack up 20 climatic variables of the study area 16](#_Toc147221119)

[Step 12. PCA and delineate seed zones 17](#_Toc147221120)

(6) [Genetic offset 22](#_Toc147221121)

[Step 13. Stack up future 20 climate distribution 22](#_Toc147221122)

[Step 14. Delineate future seed zones and calculate Euclidean distance 25](#_Toc147221123)

[Step 15. Plot the genetic offset map (Fig. 5D) 28](#_Toc147221124)

[References 29](#_Toc147221125)

# R tutorial of genomic-based niche model

Progress in niche-based models has significantly enhanced our comprehension of the intricate connections between climate variables and species distributions, leading to more precise predictions and informed decision-making in the face of rapid climate change. One essential advancement of niche models is the integration of high-resolution climate data, such as those accessible through ClimateNA, which allows for more accurate modelling at finer spatial scales. In addition, the integration of landscape genomic data has further elevated the precision and applicability of niche models in forestry applications. Here, we present a step-by-step tutorial to obtain scale-free climate data using the newly released R package ClimateNAr and construct a genomics-based niche model of lodgepole pine (*Pinus contorta*) in British Columbia and Alberta.

## (1) Species data description

### *Provenance trial*

The sampler data used in this study were collected from a raised bed common garden comprising 1,906 lodgepole pine seedlings located at Totem Field, University of British Columbia in Vancouver, BC. These seed lots originated from 281 provenances, representing the climatic range of lodgepole pine within British Columbia and Alberta (MacLachlan *et al.*, 2017).


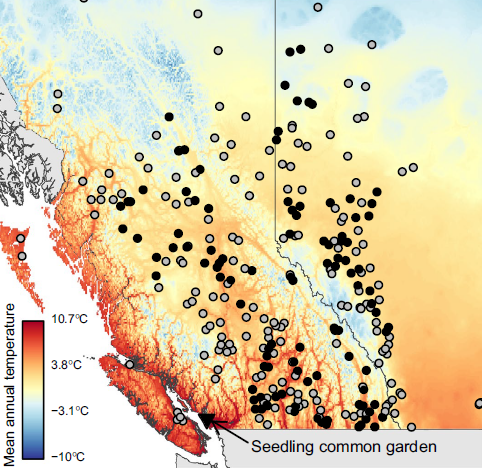


#### **Fig. S1.** Provenance of the 281 populations of lodgepole pine from which 1906 individual trees were obtained from across British Columbia and Alberta (adapted from Mahony *et al.*, 2020).

### **Genomics data**

A total of 1,906 individual trees were sampled for DNA extraction from spring needle tissue. The DNA extraction process utilized the Macherey-Nagel Nucleospin 96 Plant II Core™ Kit and was carried out using the Eppendorf EpMotion 5075™ liquid handling platform for automation. Subsequently, the samples underwent genotyping at Neogen GeneSeek (Lincoln, Nebraska) using the AdapTree lodgepole pine Affymetrix Axiom 50K SNP array specifically designed for lodgepole pine. The SNP discovery for this array was based on the lodgepole pine sequence capture data set described by Suren *et al.* (2016). The SNP array included probes for the exons of 24,388 genes and intergenic regions, with intron–exon boundaries identified by mapping the lodgepole pine transcriptome to the loblolly pine (*Pinus taeda* L.) v1.01 draft genome (Zimin *et al.*, 2014; Neale *et al.*, 2014).

The SNPs were carefully selected for inclusion, considering seedling traits, differentially expressed genes, candidate genes for climate adaptation from other conifers, mappable SNPs for a linkage map, and a set of randomly selected intergenic SNPs to control for population structure. The final SNP table for association analyses contained 32,407 SNPs, with 3,934 intergenic control SNPs specifically for population structure correction. The complete SNP data of lodgepole pine can be found at <https://adaptree.forestry.ubc.ca/> and <https://doi.org/10.1111/eva.12871>).

In this example data set, 1,906 seedlings from the Vancouver outdoor seedling common garden were genotyped, along with an additional 1,906 seedlings from the same 281 provenances grown in a separate growth chamber experiment. The median sample size for each provenance was 11 seedlings, ranging from seven to 24 (Fig. S2).


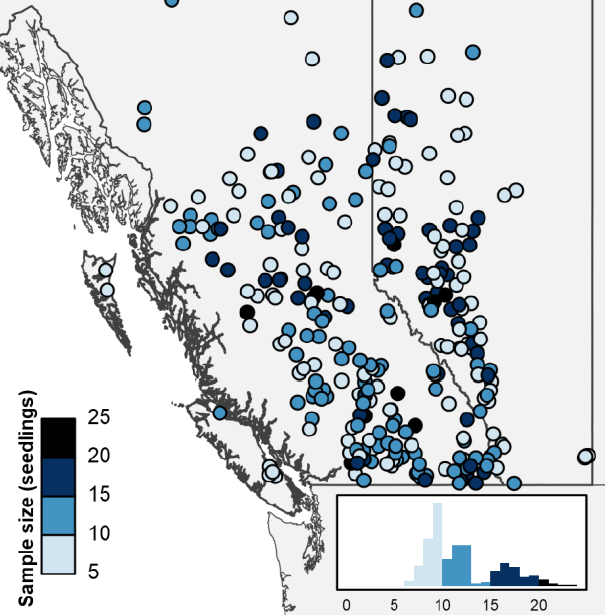


#### **Fig. S2.** Sample size of common garden genotyping. SNP array genotyping was conducted on 30 phenotyped common garden seedlings and an additional sample of seedlings grown in a growth chamber (adapted from Mahony *et al.*, 2020).

## (2) Scale-free climate data generation

### *Step 1. ClimateNA application download*

ClimateNA is a user-friendly, standalone MS Windows application designed to downscale PRISM 1971–2000 gridded monthly climate normal data with a resolution of 800 × 800 m. It transforms this data effectively into scale-free point locations. The application is equipped to calculate and derive over 200 monthly, seasonal, and annual climate variables, providing comprehensive climate information. ClimateNA not only serves as a valuable tool for present-day climate analysis but also can downscale historical and future climate variables for individual years and periods spanning from 1901 to 2100.

The coverage of ClimateNA extends across the whole of North America, making it an essential resource for climate modelling and analysis in this region. All data produced using ClimateNA are available under the CC-BY license, ensuring accessibility and unrestricted use for research and decision-making purposes. The application can be downloaded at <https://climatena.ca/>.

### *Step 2. ClimateNAr package installation*

As the “ClimateNAr” R package is not registered in CRAN, it needs to be downloaded and installed locally. The package can be downloaded at <https://register.climatena.ca/>, and can be installed locally in one of three ways:

(1) Through interface: Packages => Install package(s) from local files.

(2) Through R code: install.packages('path/ClimateNA.zip', repos=NULL, type='source'). For example: install.packages('C:/temp/climatenaAPI.zip', repos=NULL, type='source')

(3) Simply unzip the folder to the R library folder on your computer.

### *Step 3. Climate data generation for 281 locations*

#climate data obtained from ClimateNA for 20 climate variables

library(ClimateNAr)

wkDir <- "D:\\Applications\\ClimateNA_v731\\" # Application location

exe <- "ClimateNA_v7.31.exe"

inputFile <- "D:\\...\\PlSeedlots.csv"

outputFile <- "D:\\...\\PlSeedlots_1961.csv"

period <- 'Normal_1961_1990.nrm'

ClimateNA_cmdLine(exe, wkDir, period, MSY='Y', inputFile, outputFile)

We used the geographic location information of the 281 lodgepole pine provenances to generate 20 climate variables (Table S1) through the ClimateNAr package. The file name for geographic location information of the 281 lodgepole pine populations is “PlSeedlots.csv”, which is accessible in Appendix S2.

#### **Table S1.** Description of 20 climatic variables used in this document.

| **Abbreviation** | **Description** |
| --- | --- |
| MAT | Mean annual temperature (°C) |
| MWMT | Mean warmest month temperature (°C) |
| MCMT | Mean coldest month temperature (°C) |
| TD | Temperature difference between MWMT and MCMT, or continentality (°C) |
| MAP | Mean annual precipitation (mm) |
| MSP | Mean annual summer (May to September) precipitation (mm) |
| AHM | Annual heat-moisture index [(MAT + 10)/(MAP/1000)] |
| SHM | Summer heat-moisture index [MWMT/(MSP/1000)] |
| DD_0 | Degree-days below 0 °C, chilling degree-days |
| DD5 | Degree-days above 5 °C, growing degree-days |
| NFFD | Number of frost-free days |
| FFP | Frost-free period |
| bFFP | Day of the year on which FFP begins |
| eFFP | Day of the year on which FFP ends |
| PAS | Precipitation as snow (mm). For individual years, it covers the period between August in the previous year and July in the current year |
| EMT | Extreme minimum temperature over 30 years |
| EXT | Extreme maximum temperature over 30 years |
| Eref | Hargreaves reference evaporation (mm) |
| CMD | Hargreaves climatic moisture deficit (mm) |
| RH | Mean annual relative humidity (%) |

## (3) Genomic data preparation

The file name for the whole data set with all 32,407 SNPs for 1,906 seedlings is “all.Snp.csv”, which can be accessed at <https://doi.org/10.5061/dryad.56j8vq8>. As rare alleles are likely to cause false positives in further analysis, the data set was reduced by removing minor allele frequencies among all individuals < 0.05 (Gugger *et al.*, 2018). To avoid problems when building niche models, SNPs with 30% missing data across all samples are recommended to be discarded. As this data set is for demonstration only, we removed all SNPs with missing values, which left only 58 SNPs for demonstration here.

### *Step 4. Convert the raw SNP data to frequency format*

all <- read.csv('allSnp.csv');head(all);dim(all)

all0<-all[,3:32409]

## conversation

# Create an empty table with the same dimensions as 'allSnp' to store frequency values

frequency_table <- matrix(0, nrow = nrow(all0), ncol = ncol(all0))

# Iterate through each column of 'my_table'

for (col in 1:ncol(all0)) {

# Calculate the frequency of names in the current column

frequency <- table(all0[, col])

# Iterate through each cell in the current column

for (row in 1:nrow(all0)) {

# Get the name in the current cell

name <- all0[row, col]

# Calculate the frequency of the name in the current column

cell_frequency <- frequency[name]/nrow(all0)

# Assign the frequency value to the corresponding cell in the frequency table

frequency_table[row, col] <- cell_frequency

}

}

# Convert the frequency table to a data frame

frequency_df <- as.data.frame(frequency_table)

# Replace the column names of the frequency table with the original column names

colnames(frequency_df) <- colnames(all)[3:32409]

### *Step 5. Discard SNPs with minor allele frequency < 0.05*

c3 <- frequency_df

dim(c3)

snp_frequency<-cbind(all[,1:2], c3)

write.csv(snp_frequency, file = "snp_frequency.csv")

columns_to_delete <- which(colSums(c3 < 0.05) > 0)

# Remove the columns from the table

c5 <- c3[, -columns_to_delete]

# The rest snp

selected_col <- colnames(c5)

snp_9876 <- all0[,selected_col]

snp_filtered<-cbind(all[,1:2], snp_9876)

write.csv(snp_filtered, file = "snp_9876.csv")

### *Step 6. Discard the SNPs with > 30% missing data*

#### remove 30% NA values SNP ####

zero_prop <- colMeans(all=="00")

col_to_del <- names(zero_prop[zero_prop>0.3])

all_new <- all[,!names(all) %in% col_to_del]

## (4) Gradient forest model

The Gradient Forest (GF) model is a robust multivariant modelling approach well suited for constructing genomics-based niche models. It has been widely used to study the complex relationships between multiple species abundances and environmental conditions in ecological research. When building a genomics-based niche model using the GF approach, several key steps should be noted. First, the genetic data, such as SNPs, must be collected from the target species. These SNPs serve as the dependent variables, representing the genetic variation within populations. Next, relevant environmental data, including climate variables and other abiotic factors, are gathered as independent variables for the model. These environmental variables provide essential context for understanding how genetic variation relates to the distribution and performance of populations across different landscapes.

The GF model then integrates the genetic and environmental data to identify the most important environmental variables that explain allele frequency distribution patterns across the landscape. This process reveals how the genetic makeup of populations varies in response to different environmental conditions. Principal Component Analysis (PCA) or other clustering methods then can be used to group populations based on their patterns of genomic variation. This clustering facilitates the delineation of seed zones or ecotypes, which are areas where populations exhibit similar genetic characteristics and are better adapted to specific environmental conditions. Additionally, the concept of genetic offset can be applied in genomics-based niche models. Genetic offset refers to the genetic distance between current allele frequency and expected allele frequency under specific climate scenarios. This information is vital for developing effective adaptation strategies, as it provides insights into the adaptive capacity of tree populations in response to climate change. The integration of GF models with genomic data has enriched the application of niche modelling in forestry, offering a more comprehensive understanding of how genetic factors interact with the environment and influence species distributions and responses to changing climatic conditions.

### *Step 7. Merge genomic data and climate data*

op_data <- read.csv('PlSeedlots_1961.csv');head(op_data)

climate0 <- data.frame(op_data[,clmVar]);head(climate0);dim(climate0)

names(climate0)[names(climate0)=='id1']<-'ID';head(climate0)

#use the first 281 rows/locations and match with the locations in the genomic data

snp_58 <- read.csv("snp_58.csv")

rawdatafinal_A <- merge(snp_58,climate0, by = c("ID"))

write.csv(rawdatafinal_A,'snp_clm_gf_58.csv')

### *Step 8. GF model training*

library(rgdal);library(sf);library(randomForest);

library(gradientForest)

library(CEMT) #my functions

library(raster)

setwd("D:/…")

rawdatafinal_A<-read.csv('snp_clm_gf_58.csv')

dim(rawdatafinal_A)

predictor.vars <- colnames(rawdatafinal_A[,63:82]);predictor.vars

#20 climate variables

response.vars <- colnames(rawdatafinal_A[,5:62]);response.vars[1:10]

Sp_mat0<- rawdatafinal_A[,response.vars]

Sp_mat <- lapply(Sp_mat0, factor)

Sp_mat <- as.data.frame(Sp_mat)

Phys_site<- rawdatafinal_A[,predictor.vars]

nSites <- dim(Sp_mat)[1]

nSpecs <- dim(Sp_mat)[2]

lev <- floor(log2(nSites * 0.368/2))

lev

gf <- gradientForest(cbind(Phys_site, Sp_mat),

predictor.vars = colnames(Phys_site), response.vars = colnames(Sp_mat),

ntree = 200, transform = NULL, compact = T,

nbin = 201, maxLevel = lev, corr.threshold = 0.5)

gf

gf_n <- c("D:/…/gf_rst.Rdata")

save(gf,file=gf_n)

### *Step 9. GF model output plot*

The predictor overall importance plot (Fig. 5A) shows the mean accuracy importance and the mean importance weighted by allele frequency R^2^. DD5, EMT and MAT are the most important variables affecting allele frequency of 281 populations.

plot(gf, plot.type = "O")

most_important <- names(importance(gf))[1:25]

par(mgp = c(2, 0.75, 0))

The second plot is the splits density plot (plot.type="S"), which shows binned split importance and location on each environment gradient (spikes), kernel density of splits (black lines), observations (red lines) and splits standardized by observations density (blue lines). Each distribution integrates to predictor importance. These plots show where important changes in the allele frequency are occurring along the gradient (Fig. S3).

plot(gf, plot.type = "S", imp.vars = most_important,

leg.posn = "topright", cex.legend = 0.6, cex.axis = 0.5,

cex.lab = 0.7, line.ylab = 0.9, par.args = list(mgp = c(1.5,

0.5, 0), mar = c(3.1, 1.5, 0.1, 1)))


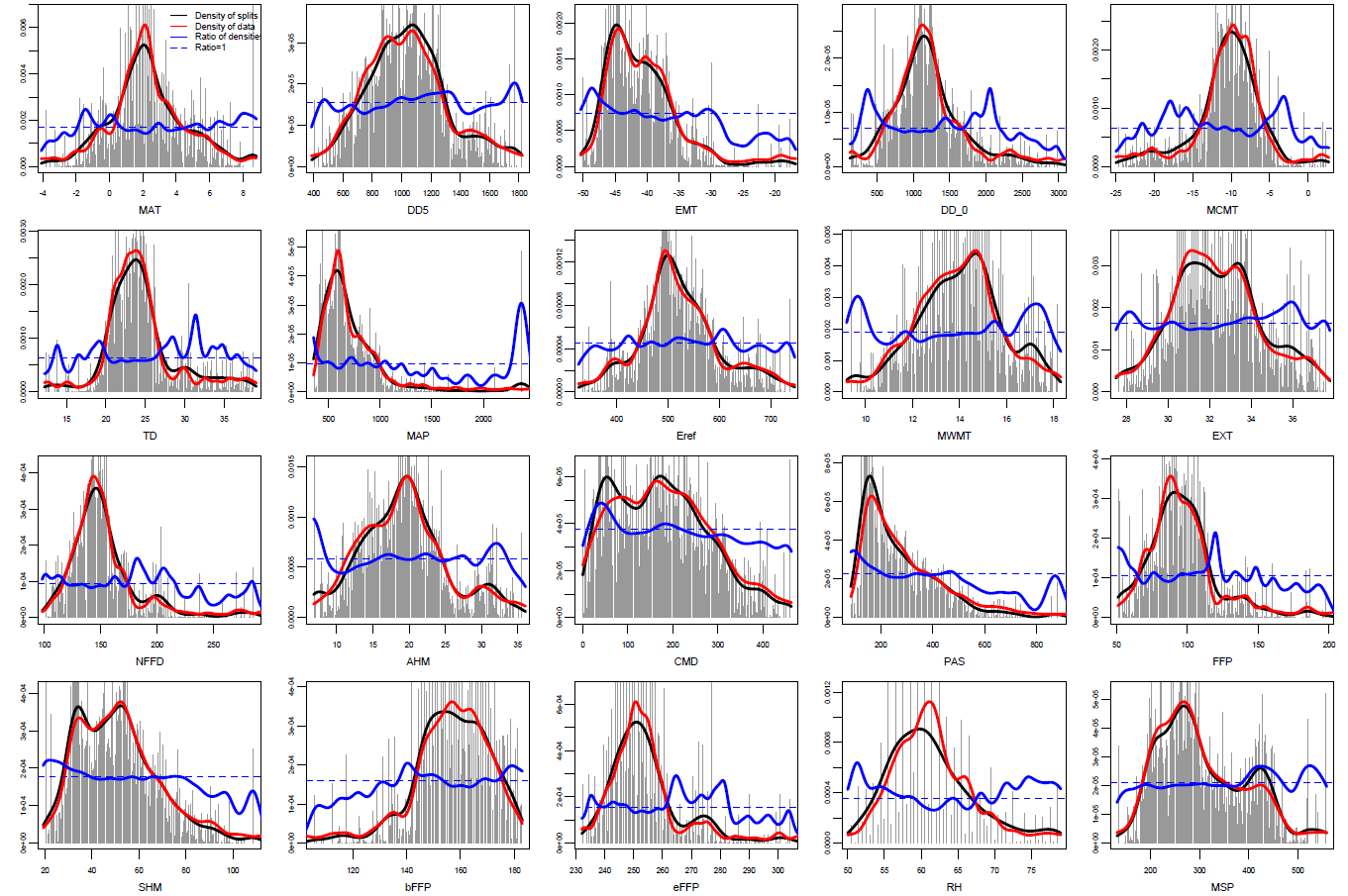


#### **Fig. S3.** Density plot showing significant allele frequency changes in all 20 climate gradients (see Table S1 for definitions of climate variables).

The third plot (Fig. S4) is the predictor cumulative plot (plot.type="C", show.species=F), which for each predictor shows cumulative importance distributions of splits improvement scaled by *R*^2^ weighted importance, and standardized by the density of observations, averaged over all SNPs. These show the cumulative change in allele frequency, where changes occur on the gradient. The fourth plot (Fig. S5) shows the *R*^2^ measure of the fit of the random forest model for each SNP.

plot(gf, plot.type = "C", imp.vars = most_important,

show.overall = F, legend = T, leg.posn = "topleft",

leg.nspecies = 5, cex.lab = 0.7, cex.legend = 0.4,

cex.axis = 0.6, line.ylab = 0.9, par.args = list(mgp = c(1.5,

0.5, 0), mar = c(2.5, 1, 0.1, 0.5), omi = c(0,0.3, 0, 0)))


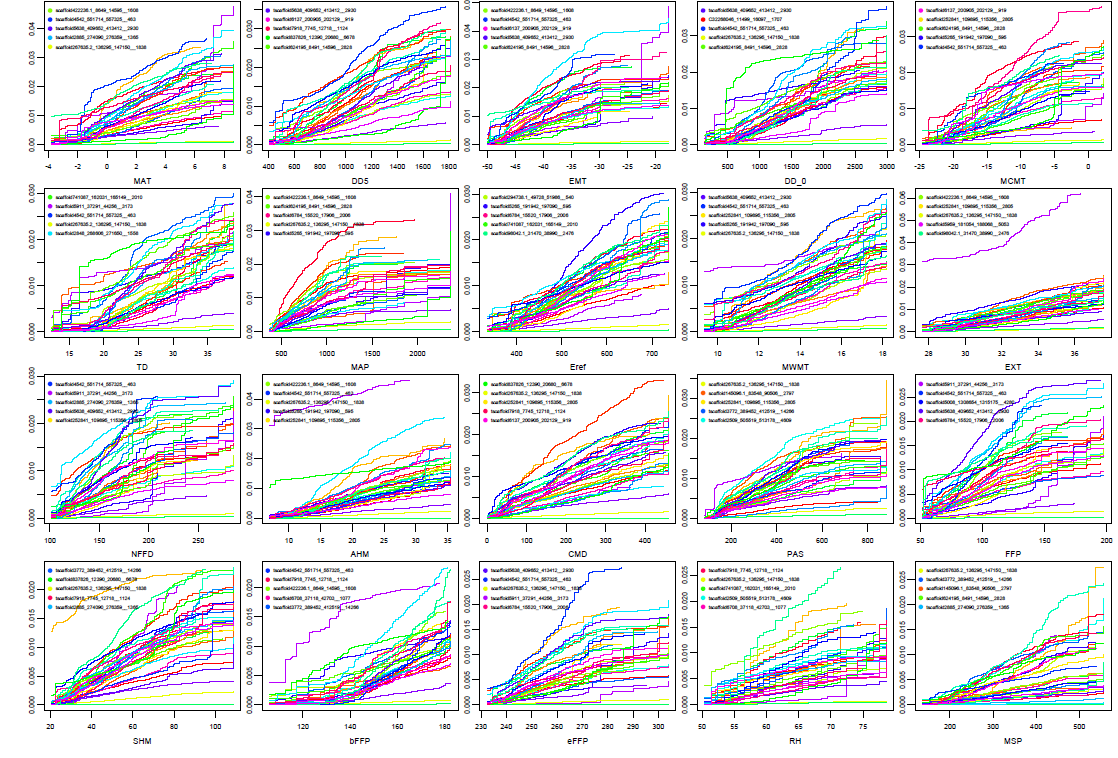


#### **Fig. S4.** Cumulative importance distributions of splits improvement scaled by *R*^2^ weighted importance for all SNPs. See Table S1 for definitions of climate variables.

plot(gf, plot.type = "C", imp.vars = most_important,

show.species = F, common.scale = T, cex.axis = 0.6,

cex.lab = 0.7, line.ylab = 0.9, par.args = list(mgp = c(1.5,

0.5, 0), mar = c(2.5, 1, 0.1, 0.5), omi = c(0, 0.3, 0, 0)))


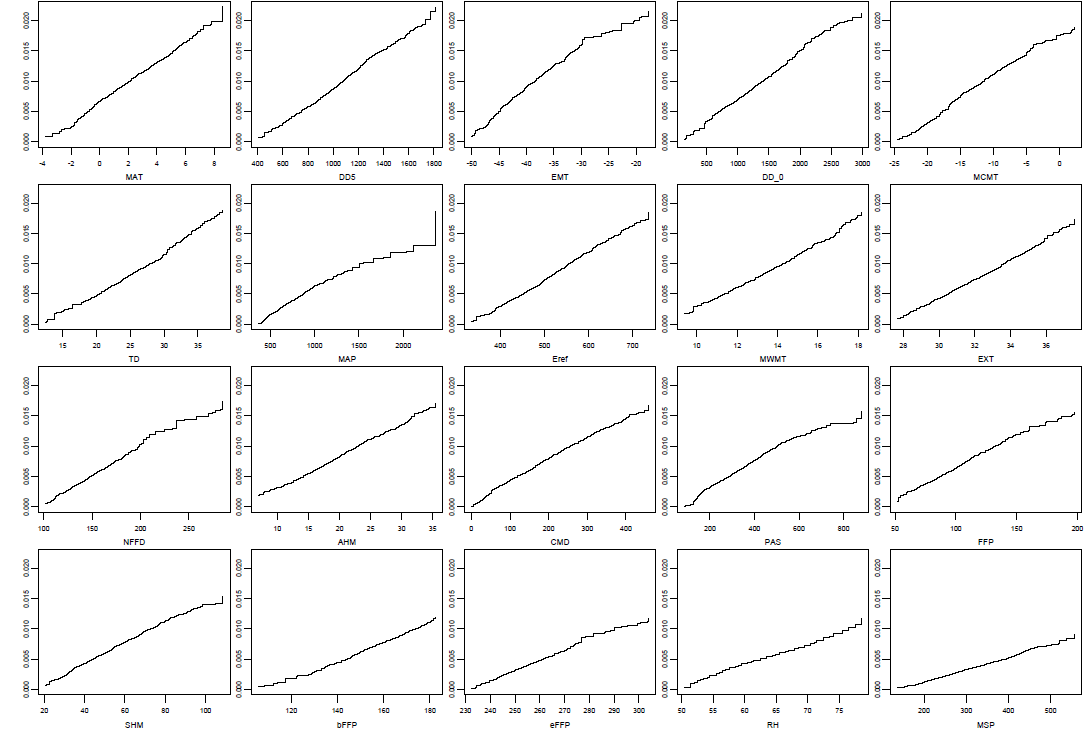


#### **Fig. S5.** Cumulative importance distributions of splits improvement scaled by *R*^2^ weighted importance for each SNP. See Table S1 for definitions of climate variables.

plot(gf, plot.type = "P",main=" ",

show.names = F, horizontal = F,

cex.axis = 1, cex.labels = 0.7, line = 2.5)

plot(gf, plot.type = "P", show.names = T, horizontal = F,

cex.axis = 1, cex.labels = 0.7, line = 2.5)

plot(gf, plot.type = "P", show.names = F, horizontal = T,

cex.axis = 1, cex.labels = 0.6, line = 2.5)

plot(gf, plot.type = "P", show.names = T, horizontal = T,

cex.axis = 1, cex.labels = 0.6, line = 2.5)

## (5) Geographic plotting

The file name for the range of lodgepole pine in British Columbia and Alberta is “pc_range.shp”, which is accessed in Appendix S3. The outline for British Columbia and Alberta is “ABBC_outline.shp”, which is accessed in Appendix S4. The datum we used for projecting the map is NAD83.

### *Step 10. Map projection*

#Load outlines

range <- readOGR("D:/…/pc_range.shp")

plot(range)

range2 <- spTransform(range, CRS("+proj=aea +lat_1=50 +lat_2=58.5 +lat_0=45 +lon_0=-126 +x_0=1000000 +y_0=0 +datum=NAD83

+units=m +no_defs +ellps=GRS80 +towgs84=0,0,0"))

plot(range2) #distribution outline with projection

#British columbia and Alberta (ABBC) outline

outline <- readOGR("D:/…/ABBC_outline.shp")

plot(outline) # ABBC outline with NO projection

outlineABBC <- spTransform(outline, CRS("+proj=aea +lat_1=50 +lat_2=58.5 +lat_0=45 +lon_0=-126 +x_0=1000000 +y_0=0 +datum=NAD83

+units=m +no_defs +ellps=GRS80 +towgs84=0,0,0"))

plot(outlineABBC) #ABBC outline with projection


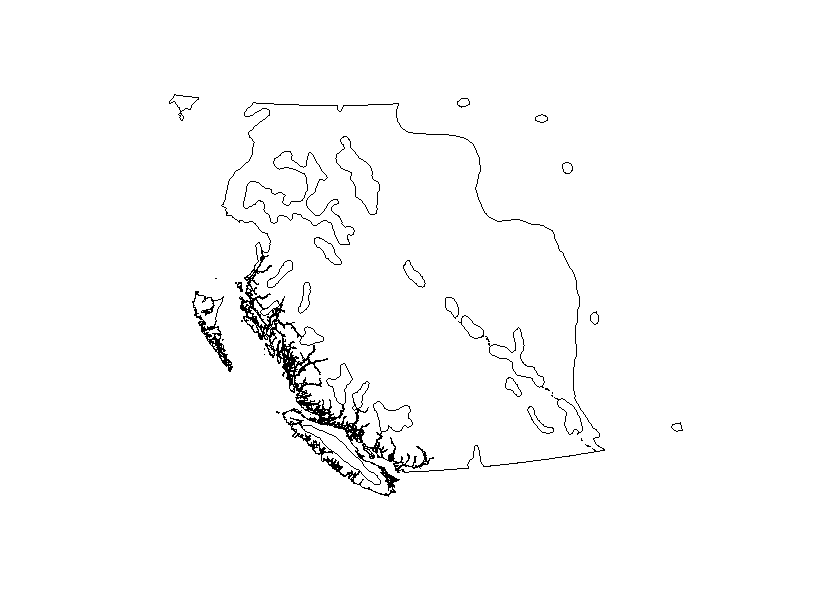


#### **Fig. S6.** The projected range of lodgepole pine in British Columbia and Alberta.


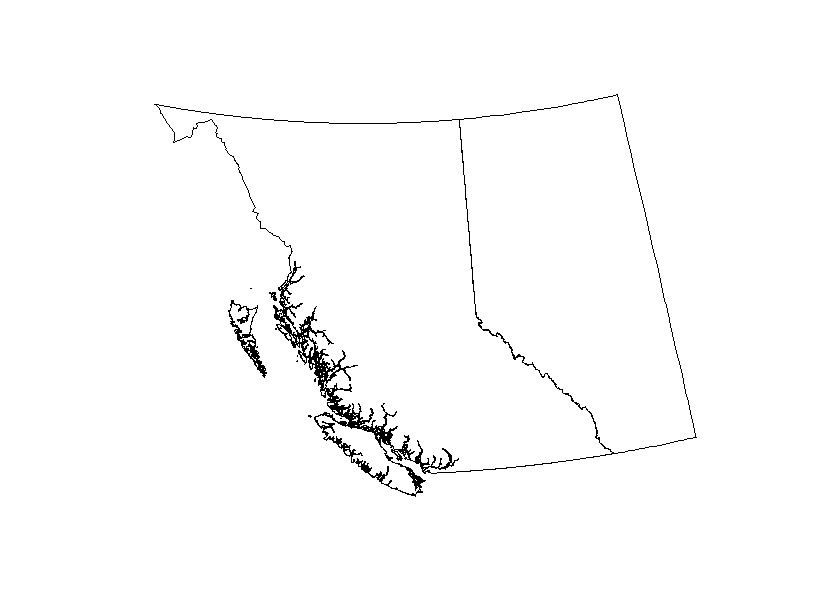


#### **Fig. S7.** The projected outlines of British Columbia and Alberta.

We used ClimateNA to generate 20 climate distribution maps of British Columbia and Alberta for the reference period (1961–1990). The resolution is 800 m × 800 m and the format is an .asc file (it can be in .tif format instead, we used a function ‘toStk3’ to determine its format). We used MAT as a demonstration (Fig. S8).

### *Step 11. Stack up 20 climatic variables of the study area*

#Function to read tif or .asc files

toStk3 <- function(x,varList,rType='tif',vConvert=F){

library(raster)

for(var in varList){

if(rType=='asc'){inF <- paste0(x,'/',var, '.asc')}

r <- raster(inF)

if(vConvert==T){

if(names(r)=='MAT'|names(r)=='MWMT'|names(r)=='MCMT'|names(r)=='TD'|names(r)=='EMT'|names(r)=='EXT'|names(r)=='AHM'|names(r)=='SHM'|names(r)=='MAR'){r <- r/10}

}

if(var==varList[1]){stk=r} else{stk=stack(stk,r)}

}

return(stk)

}

#Define list of 20 climate variables

varList <- c("MAT", "MWMT", "MCMT", "TD", "MAP", "MSP", "AHM", "SHM", "DD_0", "DD5", "NFFD", "FFP",

"bFFP", "eFFP","PAS", "EMT", "EXT", "Eref", "CMD", "RH") # For climate variables descriptions, see Table 1.

wd <- 'D:/…/Normal_1961_1990Y'

#Load climate data using the function

stk_abbc2 <- toStk3(wd,varList,rType='asc',vConvert=T)

stk_abbc2

#assign crs

crs(stk_abbc2) <- "+proj=longlat +datum=WGS84 +no_defs +ellps=WGS84 +towgs84=0,0,0"

stk_abbc2

#crop the ABBC area out with the NONE projected outline

stk_abbc3 <- crop(stk_abbc2, extent(range))

stk22 <- mask(stk_abbc3, range)

crs(stk22) <- "+proj=longlat +datum=WGS84 +no_defs +ellps=WGS84 +towgs84=0,0,0"

plot(stk22, 'MAT')

#add projection, final output (stk4 for ABBC)

stk44 <- projectRaster(stk22, crs ="+proj=aea +lat_1=50 +lat_2=58.5 +lat_0=45 +lon_0=-126 +x_0=1000000 +y_0=0 +datum=NAD83

+units=m +no_defs +ellps=GRS80 +towgs84=0,0,0")

stk44 #stk4 was used for ABBC historical 800m

plot(stk44, 'MAT')

plot(outlineABBC, add = TRUE,lwd = 0.005)


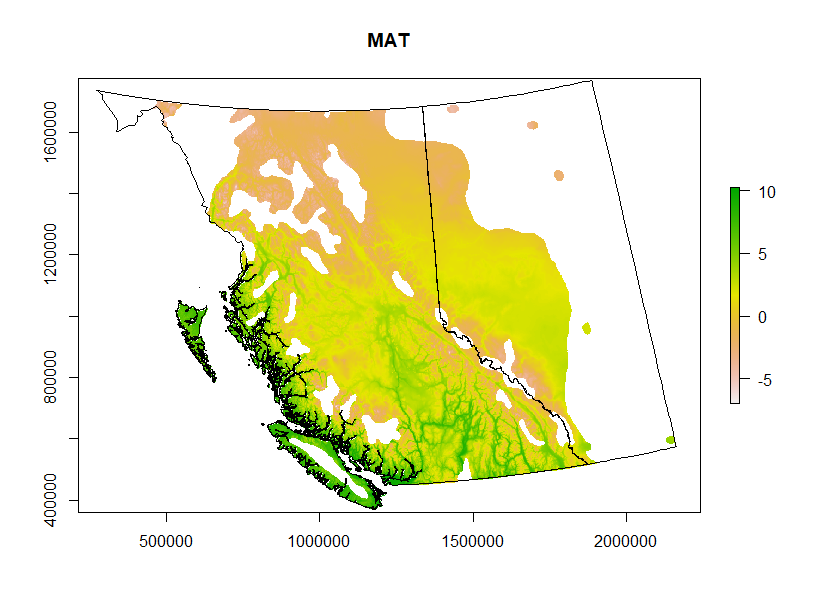


#### **Fig. S8.** The mean annual temperature (MAT) distribution in British Columbia and Alberta for the reference period 1961–1990.

One common application for genomics-based climate niche models (CNMs) is the use principal components analysis (PCA) based on SNP information and environmental variables to define seed zones (Fig. 5C). Then, we can predict the habitat range shift of the populations in different seed zones as they may have different responses to climate change.

### *Step 12. PCA and delineate seed zones*

# 12.1 Raster convert to dataframe (climate data to be predicted)

#-------------------------

csv <- rasterToPoints(stk44)

df <- as.data.frame(csv)

#write.csv(df,"ABBC_climate.csv")

#tailor the data for input

names(df)[1] <- "Longitude"

names(df)[2] <- "Latitude"

#-------------------------

# 12.2 Transform (model predict)

#-------------------------

#vars: all climate variables used in the fitted gradient forest model

df_pca <- cbind(df[,c("Longitude","Latitude")],

predict(gf,df[,varList]))

#-------------------------

# 12.3 PCA

#-------------------------

#Notes:

#vars: all climate variables used in the fitted gradient forest model

#df:climate information for all coordinates, ready to input for gf model prediction

#gf: the final fitted gradient forest model

#df_pca: a dataframe containing all GF predicted outputs, ready for PCA

PC <- prcomp(df_pca[,varList])

pcx <- PC$x

#Assign PC

pc1 <- pcx[,1]

pc2 <- pcx[,2]

pc3 <- pcx[,3]

#define RGB color palette (your choice)

r <- pc2

g <- pc3+pc1-pc2

b <- pc3-pc2

r <- (r-min(r))/(max(r)-min(r))

g <- (g-min(g))/(max(g)-min(g))

b <- (b-min(b))/(max(b)-min(b))

summary(r)

summary(g)

summary(b)

#Biplot

plot(pcx[,1:2],pch = ".", cex = 1,col = rgb(r,g,b),asp = 1)

#plot arrows (climate variables) on the biplot

vec <- c("MCMT","TD","MAP","DD_0","MAT","DD5","Eref","EMT") #Here we chose some important climate variables

lv <- length(vec)

vind <- rownames(PC$rotation) %in% vec

class(vind)

arrow1 <- PC$rotation[c(3,4,6,9,15,16,18,19),1]

arrow2 <- PC$rotation[c(3,4,6,9,15,16,18,19),2]

arrow_scale <- 20 #set a scale for the length of the arrow

plot(pcx[,1:2],pch = ".", cex = 1,col = rgb(r,g,b),asp = 1.3)

arrows(rep(0,lv),rep(0,lv),arrow1*2/arrow_scale, arrow2*2/arrow_scale,length = 0.0625)

jit <- 0.01 #distance between the text to the arrow

text(arrow1*2/arrow_scale+jit*sign(arrow1),arrow2*2/arrow_scale+jit*sign(arrow2), labels = vec)

#-------------------------

# 12.4 Map spatial pattern of predicted genomic composition

#-------------------------

#Plot spatail map with the GF-predicted results

plot(df_pca[,c("Longitude","Latitude")],pch = ".",cex =0.1 ,asp = 1,col = rgb(r,g,b))

plot(outlineABBC,add = TRUE, lwd = 0.005)

#=========================

# 12.5. Determine number of zones

#=========================

# Run this section in the R 4.2.1 or higher version

#because some packages does not work in R 3.5.1 version

#Load packages

library("knitr")

library("ggplot2")

library("factoextra")

library("cluster")

#This function (fviz_nbclust) find the within cluster variation

#variation stands for the within cluster variation

f <- fviz_nbclust(pcx,clara, method = "wss", k.max = 16)

variation <- f$data$y;f2

#then calculate the reduction in within cluster variation (reduc_var)

#-------------------------

# 12.6 Cluster points into zones

#-------------------------

ncl <- 6 #number of zones determined based on section 4 results

clPCs <- clara(pcx,ncl,sampsize=10000)

#set up the medoid color palette

medcolR <- r[clPCs$i.med]

medcolG <- g[clPCs$i.med]

medcolB <- b[clPCs$i.med]

summary(medcolR)

summary(medcolG)

summary(medcolB)

#-------------------------

# 12.7 GF-based seed and breeding zones

#-------------------------

plot(df_pca[,c("Longitude","Latitude")],pch = ".",

cex = 0.1,asp = 1,col = rgb(medcolR[clPCs$clustering], medcolG[clPCs$clustering], medcolB[clPCs$clustering]),

main = "", xlim = c(300000,2200000))

legend("bottomleft",as.character(seq(1,ncl)), pch=15, cex=1, col=rgb(medcolR,medcolG,medcolB))

points(df_pca[clPCs$i.med,c("Longitude","Latitude")], pch=as.character(seq(1,ncl)))

plot(outlineABBC, add = TRUE)


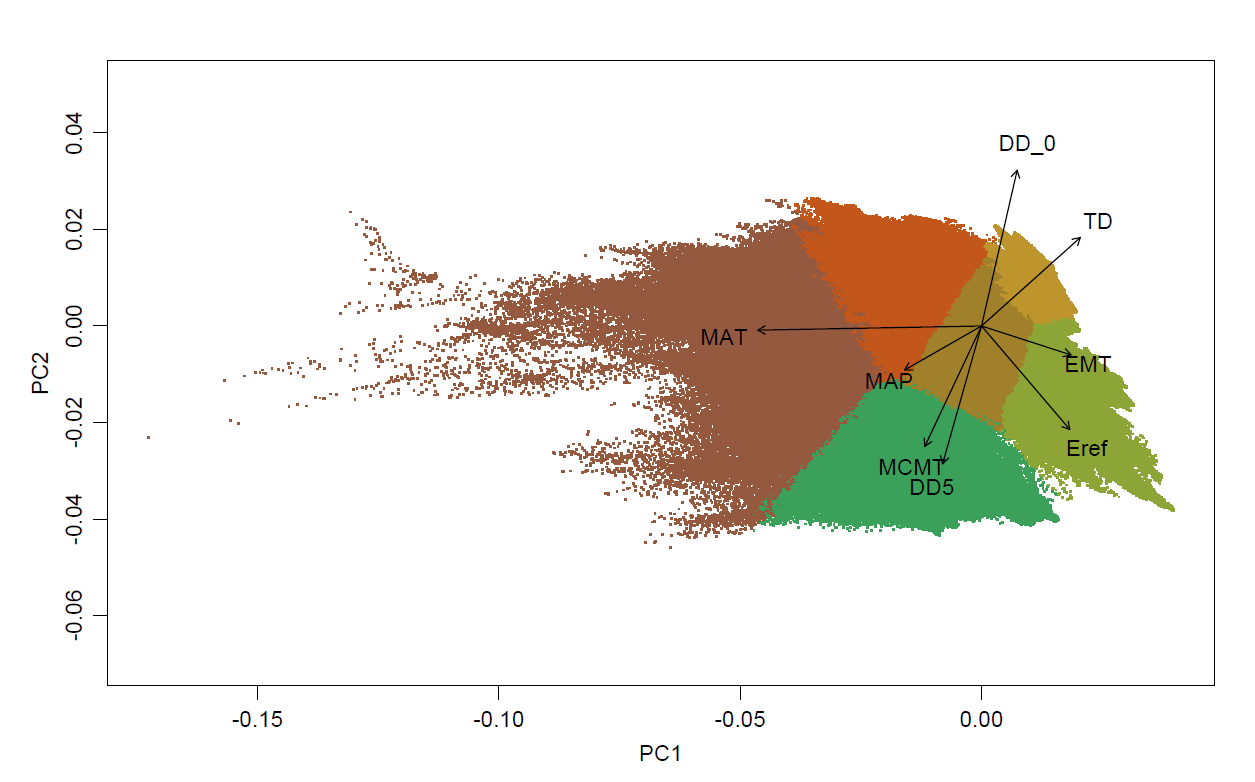


#### **Fig. S9.** The scores of two principal components for six seed zones. See Table S1 for definitions of climate variables.


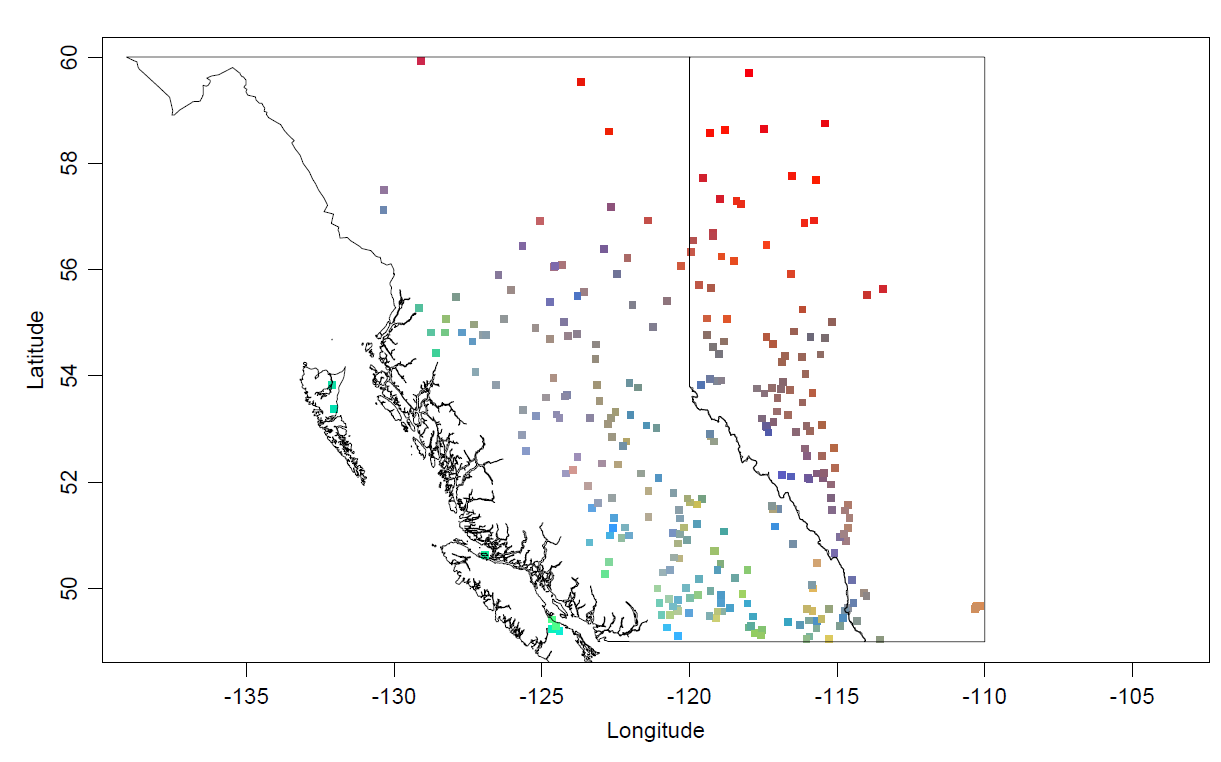


#### **Fig. S10.** The distribution of six seed zones of 281 populations.

## (6) Genetic offset

The genetic offset calculated in this example is based on a comparison between the reference period and RCP8.5 2041–2070.

### *Step 13. Stack up future 20 climate distribution*

library(raster);library(rgdal);library(sf)

library(CEMT);library(ggplot2)

library(gradientForest)

#Function to read tif files

toStk3 <- function(x,varList,rType='tif',vConvert=F){

library(raster)

for(var in varList){

if(rType=='asc'){inF <- paste0(x,'/',var, '.asc')}

r <- raster(inF)

if(vConvert==T){

if(names(r)=='MAT'|names(r)=='MWMT'|names(r)=='MCMT'|names(r)=='TD'|names(r)=='EMT'|names(r)=='EXT'|names(r)=='AHM'|names(r)=='SHM'|names(r)=='MAR'){r <- r/10}

}

if(var==varList[1]){stk=r} else{stk=stack(stk,r)}

}

return(stk)

}

#Define list of 20 climate variables

varList <- c("MAT", "MWMT", "MCMT", "TD", "MAP", "MSP", "AHM", "SHM", "DD_0", "DD5", "NFFD", "FFP", "bFFP", "eFFP","PAS", "EMT", "EXT", "Eref", "CMD", "RH") # For climate variables descriptions, see Table 1.

#RCP 8.5 (2050)

wd <- 'D:/…/15GCM-Ensemble_rcp85_2055Y'

stk_f <- toStk3(wd,varList,rType='asc',vConvert=T)

stk_f

crs(stk_f) <- "+proj=longlat +datum=WGS84 +no_defs +ellps=WGS84 +towgs84=0,0,0"

stk_f

range <- readOGR("D:/…/pc_range.shp")

plot(range)

stk_f3 <- crop(stk_f, extent(range))

stk6 <- mask(stk_f3, range);stk6

crs(stk6) <- "+proj=longlat +datum=WGS84 +no_defs +ellps=WGS84 +towgs84=0,0,0"

stk6 <- projectRaster(stk6, crs ="+proj=aea +lat_1=50 +lat_2=58.5 +lat_0=45 +lon_0=-126 +x_0=1000000 +y_0=0 +datum=NAD83

+units=m +no_defs +ellps=GRS80 +towgs84=0,0,0")

stk6

plot(stk6,"MWMT")

#### Current ####

wd0 <- 'D:/…/Normal_1961_1990Y'

#Load climate data using the function

stk_c <- toStk3(wd0,varList,rType='asc',vConvert=T)

stk_c

#assign crs

crs(stk_c) <- "+proj=longlat +datum=WGS84 +no_defs +ellps=WGS84 +towgs84=0,0,0"

stk_c

#crop the ABBC area out with the NONE projected outline

stk_abbc3 <- crop(stk_c, extent(range))

stk22 <- mask(stk_abbc3, range)

crs(stk22) <- "+proj=longlat +datum=WGS84 +no_defs +ellps=WGS84 +towgs84=0,0,0"

plot(stk22, 'MAT')

#add projection, final output (stk4 for ABBC)

stk44 <- projectRaster(stk22, crs ="+proj=aea +lat_1=50 +lat_2=58.5 +lat_0=45 +lon_0=-126 +x_0=1000000 +y_0=0 +datum=NAD83

+units=m +no_defs +ellps=GRS80 +towgs84=0,0,0")

stk44 #stk4 was used for ABBC historical 800m

plot(stk44, 'MAT')

outline <- readOGR("D:/…/ABBC_outline.shp")

plot(outline) # ABBC outline with NO projection

outlineABBC <- spTransform(outline, CRS("+proj=aea +lat_1=50 +lat_2=58.5 +lat_0=45 +lon_0=-126 +x_0=1000000 +y_0=0 +datum=NAD83

+units=m +no_defs +ellps=GRS80 +towgs84=0,0,0"))

plot(outlineABBC) #ABBC outline with projection

plot(outlineABBC, add = TRUE,lwd = 0.005)


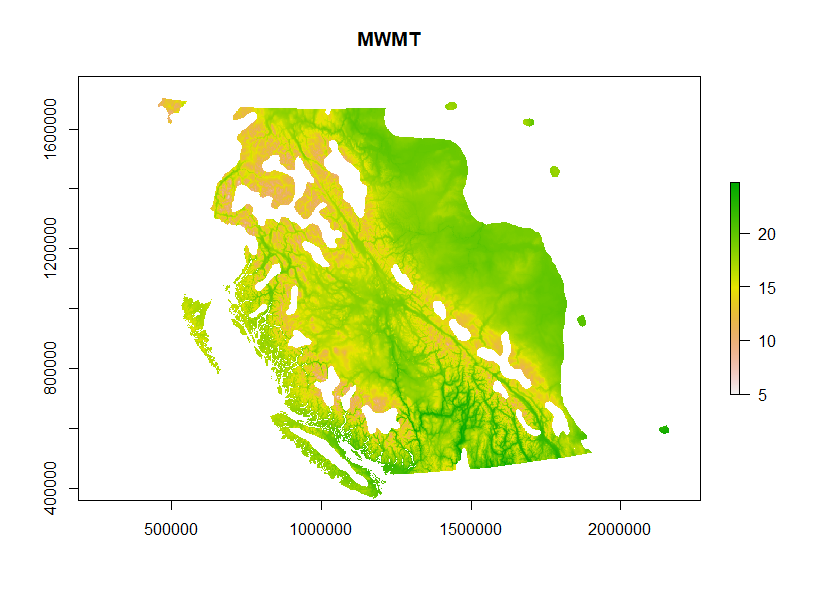


#### **Fig. S11.** Mean warmest month temperature (MWMT) distribution in British Columbia and Alberta for scenario RCP8.5 2041–2070.

### *Step 14. Delineate future seed zones and calculate Euclidean distance*

#--------------Raster into dataframe----------------

predictor.vars <- varList

#put raster into one=== (rcp 8.5)

stk_f5 <- raster::stack(stk44, stk6)

stk_f5

csv5 <- rasterToPoints(stk_f5);hd(csv5)

df5 <- as.data.frame(csv5,na.rm= TRUE);hd(df5)

names(df5)[1] <- "Longitude"

names(df5)[2] <- "Latitude"

#change all names

names(df5)[3:22] <- predictor.vars

names(df5)[23:42] <- predictor.vars

hd(df5)

gf_n <- c("D:/…/gf_rst.Rdata")

load(gf_n)

#historical

now <- cbind(df5[,c("Longitude", "Latitude")],

predict(gf,df5[,3:22]))

hd(now)

summary(now)

#(rcp45)

proj45 <- cbind(df5[,c("Longitude", "Latitude")],

predict(gf,df5[,23:42]))

hd(proj45)

summary(proj45)

proj <- proj45

#=========calculate euclidean distance===============

Offset <- sqrt(

(proj[,3]-now[,3])^2+

(proj[,4]-now[,4])^2+

(proj[,5]-now[,5])^2+

(proj[,6]-now[,6])^2+

(proj[,7]-now[,7])^2+

(proj[,8]-now[,8])^2+

(proj[,9]-now[,9])^2+

(proj[,10]-now[,10])^2+

(proj[,11]-now[,11])^2+

(proj[,12]-now[,12])^2+

(proj[,13]-now[,13])^2+

(proj[,14]-now[,14])^2+

(proj[,15]-now[,15])^2+

(proj[,16]-now[,16])^2+

(proj[,17]-now[,17])^2+

(proj[,18]-now[,18])^2+

(proj[,19]-now[,19])^2+

(proj[,20]-now[,20])^2+

(proj[,21]-now[,21])^2+

(proj[,22]-now[,22])^2)

hd(Offset)

summary(Offset)

#Uptill here, it is a list of numbers, need to regain coordinates

#=========plot the vlunerbility map (DO NOT NORMALIZE!)================

#normalize OFFSET values

min <- min(Offset, na.rm = TRUE);min

max <- max(Offset, na.rm = TRUE);max

norm_off <- (Offset-min)/(max-min); summary(norm_off)

hd(norm_off)

boxplot(norm_off)

hist (norm_off)

#----make new data frame-----

map45 <- proj[,1:2]

map45$offset <- Offset

hd(map45)

write.csv(map45, "offset_rcp85.csv")

#----points into raster-----

#library(raster)

map45r <- rasterFromXYZ(map45)


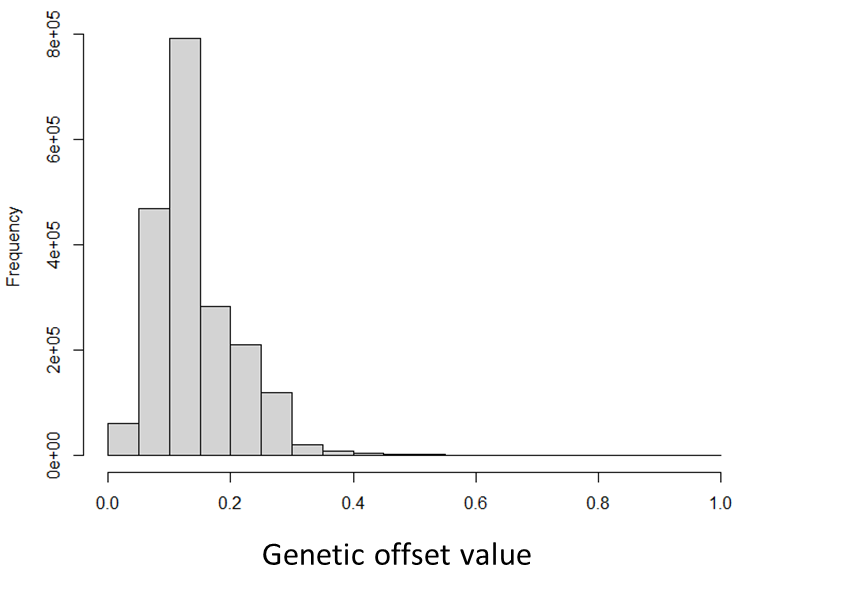


#### **Fig. S12.** Histogram plot of genetic offset values between the reference period and climate scenario RCP8.5 2041–2070.

### *Step 15. Plot the genetic offset map (Fig. 5D)*

#===========plot the maps =========================

require('RColorBrewer')

cuts = c(0.1,0.2,0.3,0.4,0.5)

pal <- colorRampPalette(rev(brewer.pal(5,'Spectral')))

display.brewer.pal(5,'Spectral')

my <- extent(200000,2250000, 32000, 1774239)

plot(my, col=NA)

plot(map45r,add = TRUE, breaks = cuts, col = pal(5),legend = F, main = "RCP8.5")

plot(outlineABBC, add = TRUE,lwd = 0.005)

#information on the plot

iwant <- hist(map45[,3], breaks = c(0,0.01,0.015,0.02,0.025,0.03,0.05))

iwant$density

iwant$counts

#===========plot the maps (2021MAY)============================

mycolor <- rev(c("darkred","red3","darkorange3", "gold2","forestgreen","darkgreen"))

library(sf)

my <- extent(200000,2250000, 32000, 1774239)

plot(my, col=NA)

plot(map45r,add = TRUE, breaks = cuts, col = mycolor,legend = F, main = "RCP4.5")

plot(outlineABBC, add = TRUE,lwd = 0.005)

#This is for generating the legend

#plot(map45r,breaks = cuts, col = mycolor,legend = T, main = "RCP4.5")

polygon_df <- st_as_sf(outlineABBC) %>% st_sf()

p <- ggplot() +

geom_raster(data = map45, aes(x = Longitude, y = Latitude, fill = offset)) +

geom_sf(data = polygon_df, fill = "transparent", color = "black", size = 0.5)+

theme(panel.background = element_blank(),

panel.grid = element_blank()) +

scale_fill_gradient(low = "lightblue", high = "purple") +

labs(x = "Longitude", y = "Latitude", fill = "Genetic offset")

p

#outF <- c("D:/…/offset85.tif");outF

ggsave("fig_offset_final.png", p, width = 6, height = 4, dpi = 300)

# References

Gugger, P. F., Liang, C. T., Sork, V. L., Hodgskiss, P. & Wright, J. W. (2018). Applying landscape genomic tools to forest management and restoration of Hawaiian koa (Acacia koa) in a changing environment. *Evolutionary Applications* **11**(2), 231–242.

MacLachlan, I. R., Wang, T., Hamann, A., Smets, P. & Aitken, S. N. (2017). Selective breeding of lodgepole pine increases growth and maintains climatic adaptation. *Forest Ecology and Management* **391**, 404–416.

Mahony, C. R., MacLachlan, I. R., Lind, B. M., Yoder, J. B., Wang, T. & Aitken, S. N. (2020). Evaluating genomic data for management of local adaptation in a changing climate: A lodgepole pine case study. *Evolutionary Applications* **13**(1), 116–131.

Neale, D. B., Wegrzyn, J. L., Stevens, K. A., Zimin, A. V., Puiu, D., Crepeau, M. W., Cardeno, C., Koriabine, M., Holtz-Morris, A. E. & Liechty, J. D. (2014). Decoding the massive genome of loblolly pine using haploid DNA and novel assembly strategies. *Genome Biology* **15**, 1–13.

Suren, H., Hodgins, K., Yeaman, S., Nurkowski, K., Smets, P., Rieseberg, L. H., Aitken, S. N. & Holliday, J. A. (2016). Exome capture from the spruce and pine giga‐genomes. *Molecular ecology resources* **16**(5), 1136–1146.

Zimin, A., Stevens, K. A., Crepeau, M. W., Holtz-Morris, A., Koriabine, M., Marçais, G., Puiu, D., Roberts, M., Wegrzyn, J. L. & de Jong, P. J. (2014). Sequencing and assembly of the 22-Gb loblolly pine genome. *Genetics* **196**(3), 875–890.
